# Recent Advances in mRNA Delivery Systems for Cancer Therapy

**DOI:** 10.1002/advs.202417571

**Published:** 2025-05-20

**Authors:** Zheng Zhang, Ya‐Nan Fan, Si‐Qi Jiang, Ya‐Jing Ma, Yao‐Ru Yu, Yu‐Xin Qing, Qian‐Ru Li, Yi‐Lin Liu, Song Shen, Jun Wang

**Affiliations:** ^1^ School of Biomedical Sciences and Engineering, South China University of Technology Guangzhou International Campus Guangzhou 511442 P. R. China; ^2^ National Engineering Research Center for Tissue Restoration and Reconstruction South China University of Technology Guangzhou 510006 P. R. China

**Keywords:** mRNA, biomaterials, cancer immunotherapies, cancer treatments, delivery systems

## Abstract

mRNA therapy is a promising approach in oncology, offering innovative applications such as tumor vaccines, protein replacement therapy, cell therapy, and gene therapy. However, challenges such as mRNA stability and delivery efficiency must be addressed. Advances in delivery system technologies are crucial for precise mRNA delivery, enhancing treatment safety and efficacy. The development of delivery systems requires accurate organ or cell targeting, intelligent release mechanisms, and optimized administration routes. This review outlines the applications of mRNA therapy in oncology, as well as the utilization of nonviral vectors, encompassing organic, inorganic, and biomimetic systems. It further elucidates the strategies for passive and active vector targeting and examines recent advances in the realm of stimuli‐responsive delivery systems that are sensitive to pH and ultrasound. Additionally, the review addresses the development of noninvasive mRNA delivery systems designed for oral and pulmonary administration. The current challenges and emerging trends of mRNA therapy are discussed, and the potential strategies to mitigate these issues are emphasized.

## Graphical abstract

This review systematically investigates the applications of mRNA therapy in cancer treatment, with particular emphasis on nonviral delivery systems, targeting strategies, stimulus‐responsive systems, and local delivery methods. Concluding with a meticulous evaluation, the review sheds light on the prevailing challenges while illuminating promising future opportunities to propel advancements in mRNA‐based cancer therapeutics.

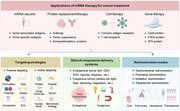

## 1 Introduction

Over the past several decades, mRNA therapy has emerged as a groundbreaking therapeutic approach, exhibiting substantial potential across various medical domains, including infectious disease vaccines, cancer immunotherapy, protein replacement therapy, and gene editing.^[^ ^1^ ^]^ Messenger RNA (mRNA), transcribed from DNA templates, serves as a carrier of genetic information, essential for protein synthesis.^[^ ^2^ ^]^

In principle, mRNA‐based techniques can regulate the synthesis of any protein or peptide by engaging the cellular machinery for protein synthesis. Compared to conventional DNA‐based therapeutics, mRNA is not integrated into the host genome, mitigating the risks associated with insertional mutagenesis and carcinogenesis.^[^ ^3^ ^]^ Furthermore, in contrast to conventional protein and peptide pharmaceuticals, mRNA enables the sustained expression of the encoded protein or peptide through continuous translation, offering extensive potential for treating diseases that require prolonged protein expression and enhanced therapeutic efficacy.^[^ ^4^ ^]^ The transient nature of mRNA activity enables precise temporal regulation of protein expression, providing significant flexibility and a wide range of therapeutic applications.^[^ ^5^ ^]^ Finally, mRNA synthesis is more efficient and reproducible, thereby enabling the rapid production of mRNA corresponding to target proteins with known sequences, which significantly expedites the drug development process.^[^ ^6^ ^]^

The inherent instability and immunogenicity of mRNA, as well as the complexities surrounding its delivery, have been pivotal factors impeding progress in mRNA therapeutics.^[^ ^7^ ^]^ As negatively charged, single‐stranded macromolecules with sizes ranging from ≈1000 to 15 000 bp, mRNA encounters substantial obstacles while traversing the anionic cell membrane.^[^ ^8^ ^]^ Furthermore, a significant proportion of mRNA is sequestered within the endosome after cell internalization, hindering its release into the cytoplasm for efficient translation, with less than 1 in 10 000 of the initial input mRNA reaching the cytoplasm.^[^ ^9^ ^]^ More importantly, the exogenous mRNA can induce immune responses in vivo, leading to local and systemic adverse reactions.

To achieve the desired therapeutic effects of mRNA, two significant challenges must be addressed: ensuring the nonimmunogenicity of mRNA and developing efficient mRNA delivery systems. Several mRNA delivery systems have been developed and are broadly categorized into viral and nonviral vectors. These vectors serve to protect mRNA from RNase degradation, enhance cellular uptake, and ensure targeted delivery, among other functions. Viruses vectors have naturally evolved to possess the inherent capability for cell targeting, internalization, and transfection, which renders them highly advantageous as gene carriers.^[^ ^10^ ^]^ Prominent viral vectors employed for mRNA delivery encompass adenoviruses, adeno‐associated viruses, and lentiviruses. However, using viral vectors presents several challenges, including complex manufacturing processes, high costs, cytotoxicity, high immunogenicity, and potential risks of cancer. Despite the clinical progress made, these safety concerns remain prominent and worrisome. In contrast, nonviral vectors offer promise in overcoming the limitations of viral vectors due to their ease of preparation and low immunogenicity. Among them, lipid nanoparticles (LNP) are the most developed nonviral carrier.^[^ ^11^ ^]^ During the COVID‐19 pandemic, BioNTech and Moderna effectively leveraged LNP technology to establish the BNT162b2 and mRNA‐1273 vaccines against SARS‐CoV‐2. This remarkable achievement highlights the significant advancements in mRNA therapy that have been witnessed in recent decades, thereby fostering substantial interest in mRNA therapeutics. Currently, mRNA therapeutics, enabled by LNP and other delivery systems, have demonstrated broad applications and advancements across various diseases, including infectious diseases (such as influenza virus, Zika virus,^[^ ^12^ ^]^ rabies virus,^[^ ^13^ ^]^ respiratory syncytial virus,^[^ ^14^ ^]^ etc.), diseases related to the deficiency or abnormality of functional proteins (such as cardiovascular diseases,^[^ ^15^ ^]^ cystic fibrosis,^[^ ^16^ ^]^ hemophilia,^[^ ^17^ ^]^ propionic acidemia,^[^ ^18^ ^]^ etc.), and genetic disorders (such as Duchenne muscular dystrophy,^[^ ^19^ ^]^ sickle cell disease,^[^ ^20^ ^]^ etc.).

**Figure 1:**
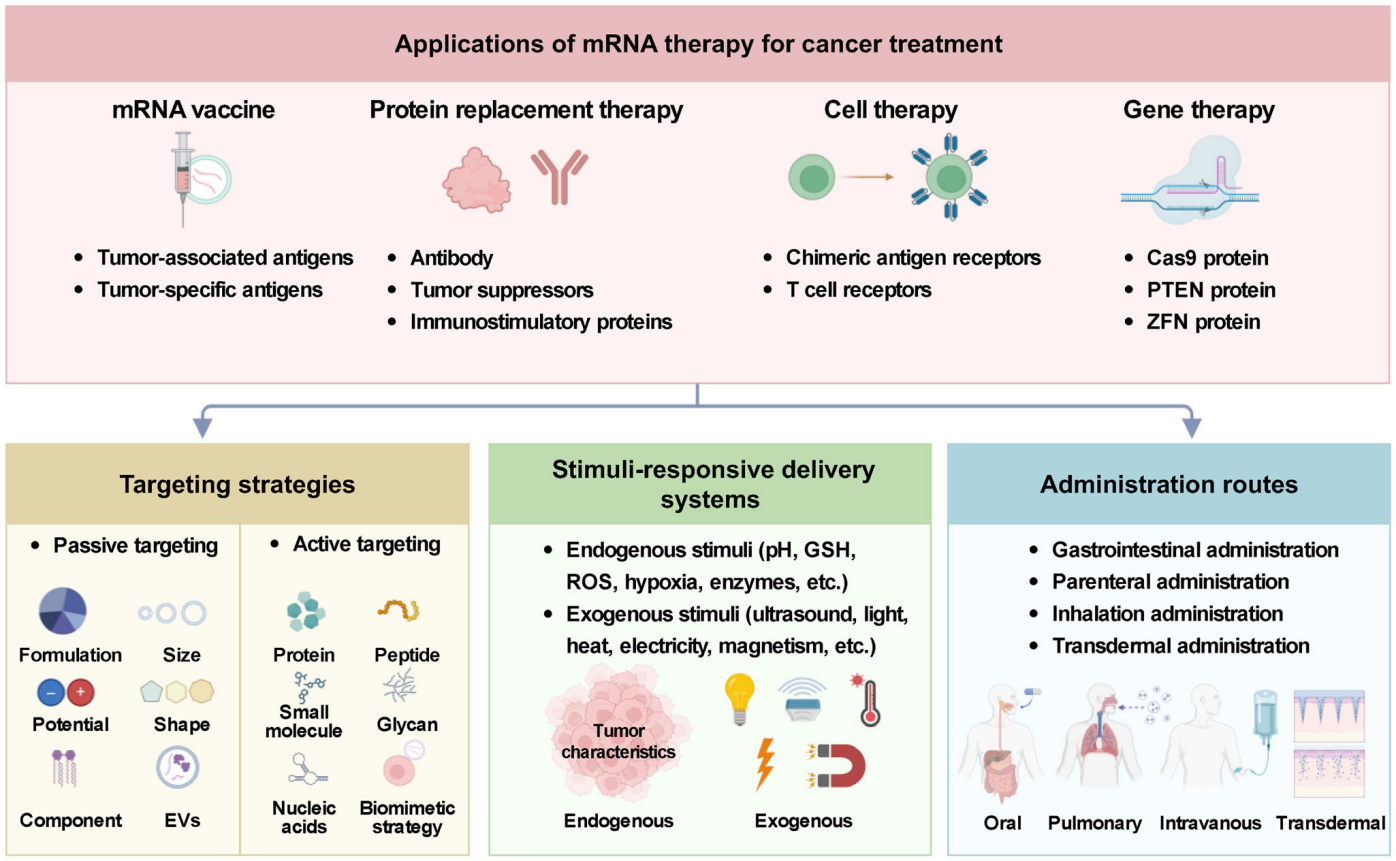
A schematic diagram elucidating the significant applications of mRNA therapy within cancer treatment, along with the targeting strategies, stimuli‐responsive delivery systems, and administration routes associated with mRNA delivery systems. Graphic created with BioRender.com.

Thus, considering the extensive body of existing research and reviews on mRNA therapy employing diverse delivery systems, we narrow our focus to mRNA‐based cancer therapies and their recent advancements. This review aims to present a comprehensive and systematic overview of the progress in mRNA‐based cancer treatment, as illustrated in **Figure** **1**. We begin by extensively discussing the diverse biomedical applications of mRNA therapy in cancer treatment, including cancer mRNA vaccines, protein replacement therapy, cell therapy, and gene therapy. Furthermore, we outline the latest advancements in nonviral delivery systems, encompassing organic, inorganic, and biomimetic delivery vectors. Additionally, we briefly explore recent advancements in targeting strategies, stimuli‐responsive delivery systems, and administration routes. The paper concludes by examining the potential challenges and opportunities for expediting progress in mRNA therapy for cancer treatments.

## 2 mRNA Therapy for Cancer Treatment

### 2.1 Cancer mRNA Vaccines

Cancer mRNA vaccines have the characteristics of broad applicability, high efficiency, safety, rapid development potential, and cost‐effective manufacturing capabilities. However, the application of mRNA vaccines was limited by instability, innate immunogenicity, and low efficiency. Proper mRNA structure modifications (e.g., codon optimization, nucleotide modification, and self‐replicating mRNA) and delivery technologies (e.g., lipid nanoparticles, polymers, and peptides) may help overcome these issues. Thus, mRNA vaccines have emerged as a promising platform for cancer immunotherapy. After vaccination, mRNA vaccines effectively express tumor antigens in antigen‐presenting cells (APCs), thereby promoting the activation of APCs and stimulating both innate and adaptive immune responses. Vaccines targeting tumor‐associated antigens (TAAs) or tumor‐specific antigens (TSAs) can specifically target and destroy malignant tumor cells that overexpress these antigens. They also leverage immune memory to induce long‐lasting therapeutic effects.^[^ ^21^ ^]^

#### 2.1.1 Tumor‐Associated Antigens

Tumor cells express a large number of TAAs, and tumor vaccines expressing different TAAs delivered by nanoparticles have been clinically tested. Indeed, the FDA has approved two preventive vaccines, one for the human papillomavirus, which causes 70% of cervical cancer virus subtypes, and another for the hepatitis B virus, which can lead to liver cancer.^[^ ^22^ ^]^ Many well‐established pharmaceutical companies and emerging biotechnology firms are actively involved in developing oncology mRNA vaccines, having made notable breakthroughs in recent years. For example, Hefei RNAlfa Biotechnology Co., Ltd. has developed AFN0328, an injectable therapeutic targeting HPV16/18‐associated premalignant lesions. The investigational new drug application for AFN0328 has been approved in China (CXSL2400288), thereby facilitating its progression to clinical evaluation. Notably, BNT111, an mRNA vaccine based on BioNTech's proprietary FixVac platform, encodes a fixed set of four melanoma‐associated antigens: NY‐ESO‐1, Tyrosinase, MAGE‐A3, and TPTE. In July 2024, phase II clinical trial data revealed that BNT111, in combination with the PD‐1 inhibitor Libtayo (Cemiplimab), significantly improved the overall response rate in patients with unresectable stage III or IV melanoma who were refractory to or had relapsed after PD‐1 therapy (NCT04526899).

#### 2.1.2 Tumor‐Specific Antigens

However, several obstacles limit the further application of TAA vaccines, including: 1) only a limited number of TAAs have been identified for certain solid tumors, leading to restricted applications; 2) the wide variation of TAAs in patients, leading to immune evasion and the generation of resistance; 3) TAAs also appear in normal tissues. Vaccines targeting TAAs may initiate central and peripheral tolerance reactions, reducing vaccination efficiency. Tumor‐specific antigens, known as neoantigens, are now the core targets of mRNA vaccines. Compared with TAAs, TSAs are more effective and can target unique tumor antigens. Neoantigens originate from random somatic mutations in tumor cells and are absent in normal cells. Neoantigens can be recognized by the host immune system as a “non‐self” motif, making them an attractive target for cancer immunotherapy.^[^ ^23^ ^]^ For example, Autogene cevumeran (BNT122) is designed based on tumor‐specific somatic mutation data obtained from each patient's tumor tissue and is encapsulated in liposomal RNA (RNA‐LPX) to stimulate T cell responses against up to 20 neoantigens. It is intended for patients with pancreatic ductal adenocarcinoma and is currently in phase II clinical trials (NCT05968326). Its phase I clinical trial data showed that pancreatic cancer patients receiving the mRNA neoantigen vaccine had significantly prolonged recurrence‐free survival, with the vaccine‐induced CD8^+^ T cell clones having an average lifespan of 7.7 years, suggesting a correlation between vaccine response and pancreatic cancer recurrence.^[^ ^24^ ^]^

### 2.2 mRNA Protein Replacement Therapy

mRNA protein replacement therapy involves in vitro transcribed mRNA to direct the translation of pharmacologically active proteins within the body. It can serve as a protein replacement for direct immunotherapies, such as antibodies and cytokines, or other protein‐based therapeutics.^[^ ^25^ ^]^ Protein therapies have demonstrated remarkable efficacy in clinical trials. Nevertheless, hurdles such as stringent quality control, purification processes, and the inherent short half‐life of antibodies have constrained their full potential. To surmount these impediments, mRNA‐based strategies for in vivo protein production have been explored. mRNA‐encoded proteins offer several distinct advantages over traditional protein therapeutics: 1) a singular in vitro transcription (IVT) mRNA design can be employed for the production and purification of multiple proteins; 2) the IVT mRNA can be readily optimized to generate improved protein variants by modifying the coding sequence; 3) the IVT mRNA leverages the host cell's translational machinery, ensuring correct protein folding and post‐translational modifications; 4) proteins produced from mRNA tend to exhibit extended serum half‐lives.^[^ ^26^ ^]^ Common mRNA‐encoding proteins include antibodies (e.g., monoclonal and bispecific antibodies),^[^ ^27^ ^]^ tumor suppressors (e.g., P53, KDM6A, PTEN),^[^ ^28^ ^]^ and immunostimulatory proteins (e.g., OX40, OX40L, IL‐12, IL‐15, IL‐23, IFN‐α).^[^ ^29^ ^]^ For example, the expression of IL‐21, IL‐7, and 4‐1BBL may be associated with improved overall survival in cancer patients. A.E.I. Hamouda et al. developed a Triplet LNP to deliver a mixture of mRNA encoding IL‐21 and IL‐7, as well as the immunostimulatory molecule 4‐1BB ligand. This approach stimulated the immune system to recognize and eliminate cancer cells while minimizing systemic exposure and toxicity.^[^ ^30^ ^]^

### 2.3 mRNA‐Based Cell Engineering

**Figure 2:**
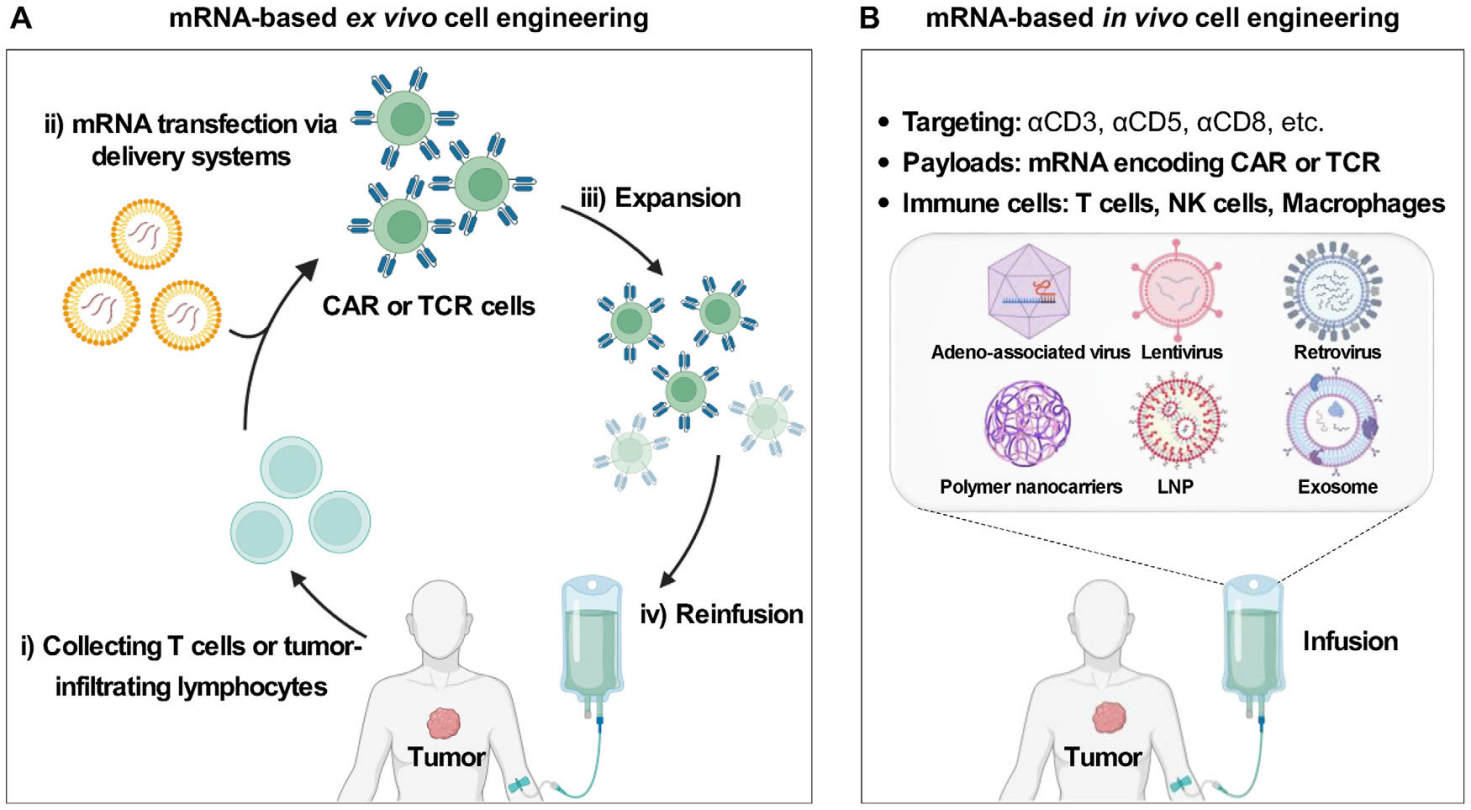
Schematic illustration showing the mRNA‐based ex vivo or in vivo cell engineering. A) The mRNA‐based ex vivo cell engineering involves cell collection, cell engineering, cell expansion, and subsequent reinfusion. B) Direct in vivo cell engineering based on cell‐targeting delivery systems encoding chimeric antigen receptors (CAR) or T cell receptors (TCR) mRNA. Reproduced with permission.^[^ ^31^ ^]^ Copyright 2024, Elsevier. Graphic created with BioRender.com.

The technology of engineering immune cells using mRNA‐encoding chimeric antigen receptors (CAR) or T cell receptors (TCR) has emerged as a new frontier in cancer therapy. Although electroporation has been the mainstream method for generating engineering cells ex vivo using mRNA, it often results in significant cell death, making it unsuitable for direct and large‐scale applications. Biomaterial‐based delivery vectors are gaining significant attention as alternatives to electroporation for mRNA‐based cell engineering, both ex vivo and in vivo (**Figure** **2**).^[^ ^31^ ^]^

#### 2.3.1 mRNA‐Based Ex Vivo Cell Engineering

In recent years, novel LNP has been developed for ex vivo delivery of CAR mRNA to immune cells. In 2020, Billingsley et al. engineered an mRNA delivery vector known as C14‐4 LNP. Treatment of primary human T cells with C14‐4 LNP carrying CAR mRNA resulted in CAR expression and antitumor activity comparable to electroporation yet significantly reduced cytotoxicity.^[^ ^32^ ^]^ In another study, Ye et al. developed 9322‐O16B and 76‐O17Se LNP for delivering modified CAR mRNA to macrophages and CD8^+^ T lymphocytes, effectively eradicating B lymphomas in vitro.^[^ ^33^ ^]^ These findings underscore the feasibility of employing mRNA‐LNP for ex vivo engineering of immune cells. Furthermore, Promab Biotechnologies has successfully transfected expanded natural killer (NK) cells with CD19‐CAR mRNA‐LNP and BCMA‐CAR mRNA‐LNP, achieving a CAR expression rate exceeding 78%. The CD19‐CAR‐NK cells exhibited superior in vivo efficacy against Nalm‐6 leukemia tumors.^[^ ^34^ ^]^ In addition to LNP, other biomaterials, such as charge‐altering releasable transporters (CART), have also been utilized to construct mRNA‐based CAR immune cells. CART BDK‐O7:N7:A13 is an oligomer with higher efficiency and lower cytotoxicity than electroporation. This oligomer can be used to deliver mRNA encoding an antihuman CD19/4‐1BB/CD3ζ CAR, effectively generating CAR‐NK cells without prior activation or phenotypic alteration of the NK cells.^[^ ^35^ ^]^ Additionally, exosomes expressing anti‐CD3/CD28 single‐chain variable fragments (scFvs) can also deliver CAR mRNA for the in vitro production of CAR‐T cells.^[^ ^36^ ^]^

#### 2.3.2 mRNA‐Based In Vivo Cell Engineering

The ex vivo production of CAR cell products is costly and laborious, necessitating intricate procedures, specialized personnel, and sophisticated equipment. To bypass these constraints, biomaterials for targeted mRNA delivery to immune cells present a promising strategy for in vivo CAR cell generation.^[^ ^37^ ^]^ In 2017, Stephan and colleagues first reported the use of poly(β‐amino ester)‐47 (PBAE‐447) based DNA nanoparticles modified with anti‐CD3e f(ab')2 fragments for in vivo T cell programming.^[^ ^38^ ^]^ In 2020, the team utilized analogous nanoparticles coated with anti‐CD8 antibodies to facilitate transient CAR or TCR mRNA expression in circulating T cells, demonstrating tumor regression through repeated administration of the nanoparticles, akin to infused ex vivo engineered CAR‐T cells.^[^ ^39^ ^]^ Yang et al. engineered liver macrophage‐targeting LNP‐carrying mRNA encoding CAR and ITIMs‐lacking CD24‐Siglec‐G (Siglec‐GΔITIMs). This approach notably enhanced macrophage phagocytosis and augmented the efficacy of HCC immunotherapy.^[^ ^40^ ^]^ Furthermore, Gu and colleagues explored 36 CAR combinations with distinct intracellular domains of macrophages, utilizing a macrophage‐targeted mRNA‐LNP system to efficiently construct CAR macrophages in situ within the body, thereby meeting the requirements for tumor immunotherapy.^[^ ^41^ ^]^

**Figure 3:**
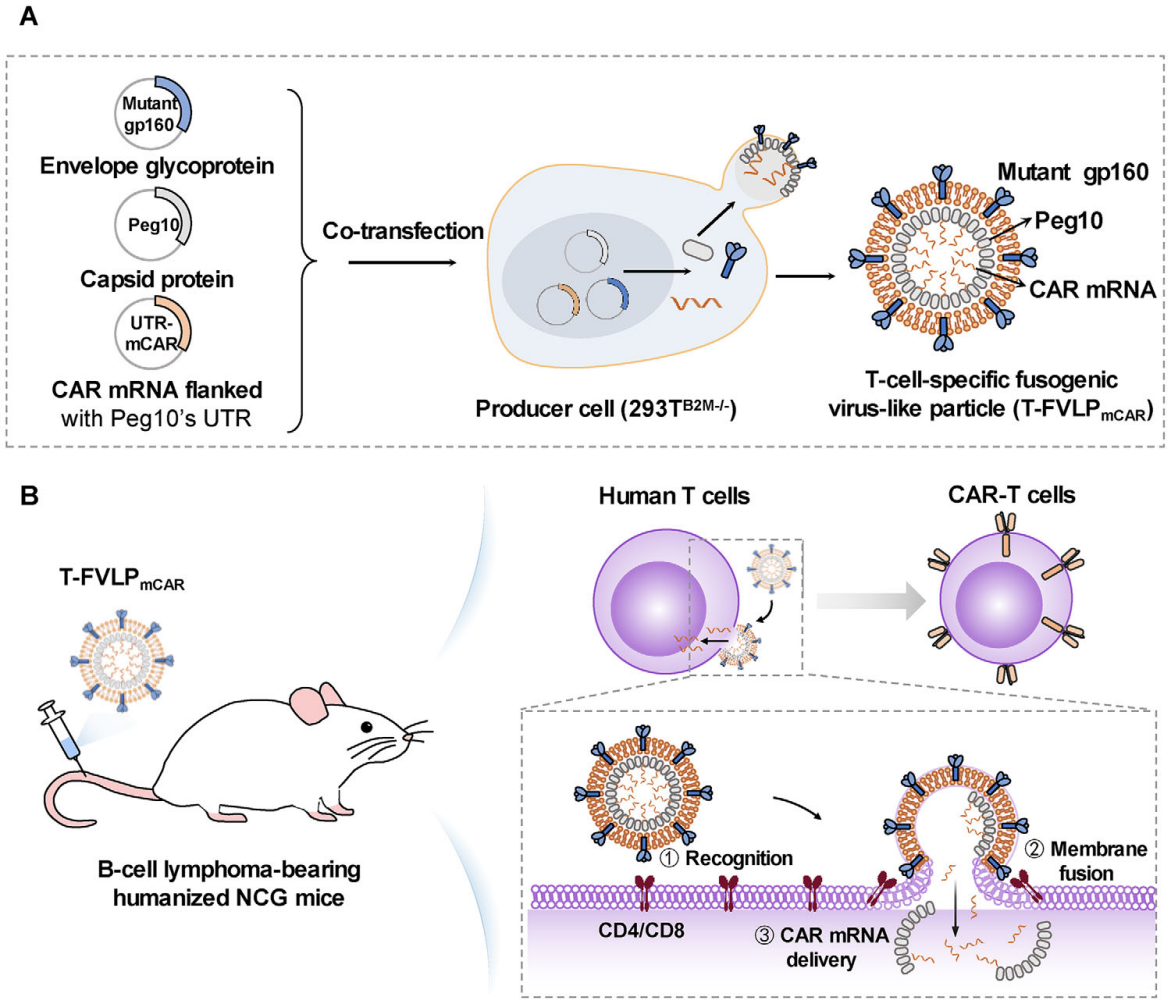
mRNA‐based T cell engineering in vivo. A) Schematic representation of T cell‐specific fusogenic virus‐like particle (T‐FVLP_mCAR_). B) The schematic shows T‐FVLP_mCAR_ recognizing and fusing with the T cell membrane for CAR mRNA delivery to generate CAR‐T cells in vivo. Reproduced with permission.^[^ ^42^ ^]^ Copyright 2025, Cell Press.

However, the limited cell specificity and efficacy of current cytoplasmic delivery systems have impeded the advancement of mRNA‐based cell therapies in vivo. Wang et al. designed a T cell‐specific fusogenic virus‐like particle (T‐FVLP_mCAR_), which displays a mutant HIV envelope glycoprotein (mutant gp160) on its surface and loads CAR mRNA via capsid protein Peg10. The mutant gp160 can specifically recognize human T cells, enabling T‐FVLP_mCAR_ to fuse with the T cell membrane and directly deliver CAR mRNA into the cytoplasm for in situ CAR‐T cell production. Results show T‐FVLP_mCAR_ converts 3.2% of circulating T cells into ahCD19 CAR‐T cells, with no CAR expression in non‐T cells. In immune humanized NCG mice with Raji‐Luc B‐cell lymphoma, T‐FVLP_mCAR_ treatment achieved 83.5% to 99.0% tumor inhibition, with some mice experiencing complete tumor regression (**Figure** **3**).^[^ ^42^ ^]^

### 2.4 Gene Therapy

Genome editing technologies can manipulate specific DNA sequences in the host genome through deletion, insertion, or alteration. Gene editing therapies have been widely applied in the treatment of cancer. The mechanisms of gene editing systems in cancer therapy primarily encompass the following aspects: 1) gene editing of the tumor‐associated gene;^[^ ^43^ ^]^ 2) inducing homology‐directed repair (HDR);^[^ ^44^ ^]^ 3) combining with chemotherapy,^[^ ^45^ ^]^ photothermal therapy,^[^ ^46^ ^]^ photodynamic therapy,^[^ ^47^ ^]^ and sonodynamic therapy;^[^ ^48^ ^]^ 4) enhancing CAR or TCR cell therapies;^[^ ^49^ ^]^ 5) modulating the tumor microenvironment.^[^ ^50^ ^]^ However, in vivo gene editing therapies offer permanent genetic changes, raising potential ethical concerns regarding human reproduction and genetics. Meanwhile, mRNA delivery mitigates off‐target effects and adverse reactions associated with transient expression and cytoplasmic translation, thereby obviating the need for nuclear delivery.

Currently, the CRISPR/Cas system is the most commonly used due to its high efficiency, versatility, and ease of operation. To achieve effective and precise cancer treatment, CRISPR‐Cas9 components must penetrate various physical barriers to reach target cells and transport Cas9 protein and sgRNA into the nucleus for gene editing. Indeed, considerable advancements have been achieved to date. For instance, Rosenblum et al. delivered Cas9 mRNA and sgPLK1 using an efficient LNP platform (cLNP), which achieved ≈70% gene editing, reduced tumor growth by 50%, and boosted survival by 30%. For disseminated tumors, cLNP was engineered to express EGFR. Then, the sgPLK1‐cLNP selectively edited genes in ovarian tumors by ≈80%.^[^ ^51^ ^]^ Siewert's team designed unimolecular dendritic lipid nanoparticles (dLNP) that encapsulate Cas9 mRNA, sgRNA, and a single‐stranded DNA template to correct the Y66H amino acid mutation. The optimized 4A3‐SC8 dLNP achieved an editing efficiency of over 91% in all cells and an HDR efficiency of over 20% in vivo within xenografted tumors.^[^ ^52^ ^]^ The team also utilized multifunctional dLNP to codeliver focal adhesion kinase (FAK) siRNA, Cas9 mRNA, and sgRNA. It demonstrated that reducing extracellular matrix stiffness can enhance gene editing in tumors by more than 10‐fold.^[^ ^53^ ^]^ Other engineered nucleases, including zinc finger nucleases (ZFN) and transcription activator‐like effector nucleases (TALEN) also could be delivered as RNA by nucleic acid delivery systems, holding great potential for cancer therapy.^[^ ^54^ ^]^

## 3 Nonviral mRNA Delivery Systems

### 3.1 Organic Delivery Systems

Utilizing nonviral vector materials with specific physicochemical properties facilitates efficient mRNA transfection. Nonviral vectors present numerous advantages, including their diverse material sources, controllable chemical structures, absence of carrier capacity limitations, and ease of large‐scale production.^[^ ^55^ ^]^

#### 3.1.1 Peptide and Protein‐Based Delivery Systems

**Figure 4:**
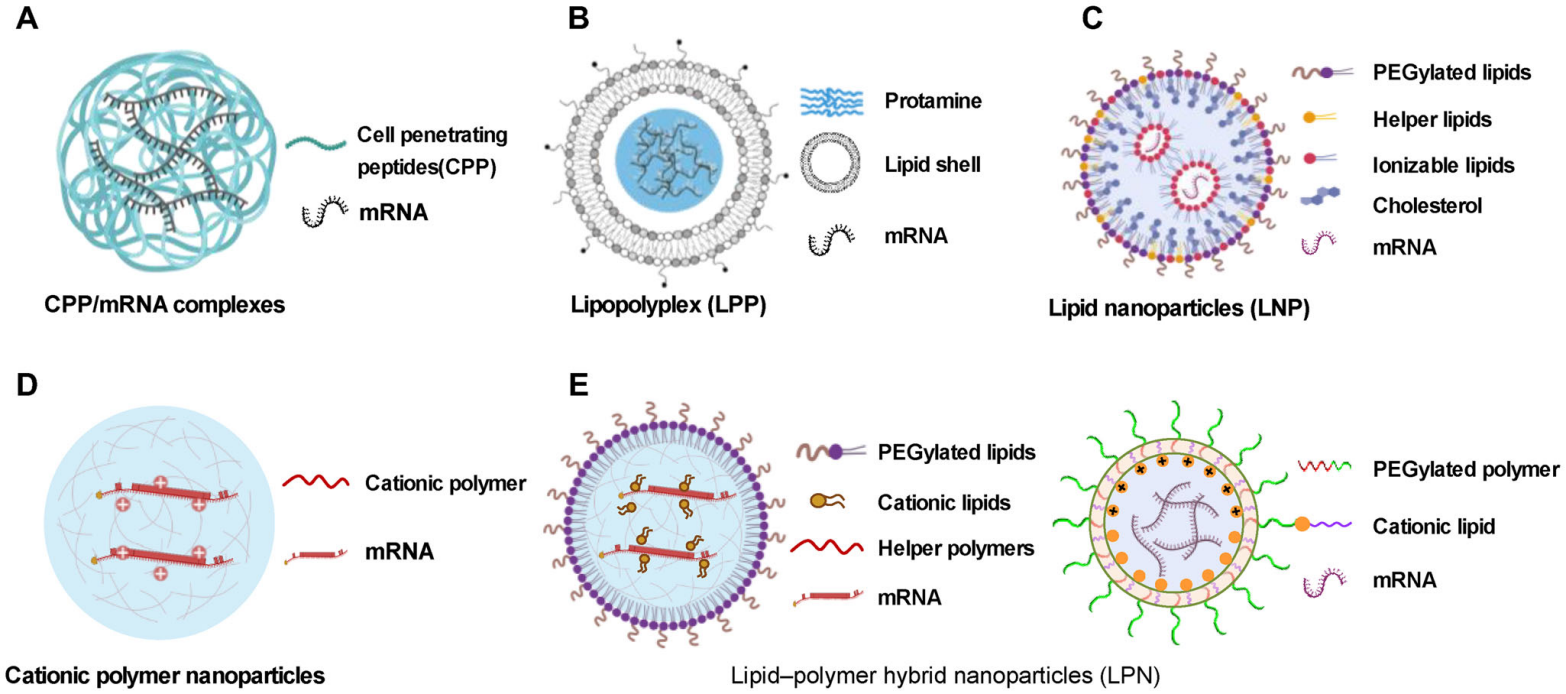
Schematic diagram of various assembled organic nanoparticles loading mRNA. A) Schematic illustration for construction of CPP/mRNA complexes. CPP, Cell‐penetrating peptides. B) Schematic diagram showing the formation process of lipopolyplex (LPP). Reproduced with permission.^[^ ^67^ ^]^ Copyright 2024, AAAS. C) Schematic diagram of typical lipid nanoparticles (LNP): PEGylated lipids (e.g., PEG‐DMG for mRNA‐1273 or ALC‐0159 for BNT162b2), ionizable lipids (e.g., SM‐102 for mRNA‐1273, ALC‐0315 for BNT162b2), helper lipids (e.g., DSPC for mRNA‐1273/BNT162b2), and cholesterol (for mRNA‐1273/BNT162b2). D) Schematic diagram of representative cationic polymers nanoparticles: cationic polymers (e.g., PBAE or hPBAE). PBAE, poly(β‐amino ester). hPBAE, hyperbranched poly(β‐amino ester). E) Schematic diagrams of lipid‐polymer hybrid nanoparticles (LPN). LPN based on liposomes (a subtype): the diagram shows the assembly of PEGylated lipids (e.g., PEG‐DSPE or PEG‐DMPE), cationic lipids (e.g., G0‐C14), and helper polymers (e.g., PLGA or PLA). Reproduced with permission.^[^ ^7^ ^]^ Copyright 2022, Springer Nature. LPNs based on polymeric nanoparticles (another subtype), known as cationic lipid‐assisted nanoparticles (CLAN): the schematic diagram illustrates the assembly of PEGylated helper polymers (e.g., PEG‐PLGA or PEG‐PLA) and cationic lipids (e.g., BHEM‐Chol or DOTAP). The graphic was created with BioRender.com.

The characteristics of peptides and proteins encompass exceptional biocompatibility, degradability, facile modification, and the ability to target actively. Cell‐penetrating peptides (CPP), which serve as potent tools for mRNA delivery, consist of 4–40 amino acids and encompass cationic, amphiphilic, and hydrophobic variants.^[^ ^56^ ^]^ These peptides could enhance mRNA nuclease stability,^[^ ^57^ ^]^ cellular penetration,^[^ ^58^ ^]^ endosomal escape efficiency,^[^ ^59^ ^]^ and cell targeting.^[^ ^60^ ^]^ Additionally, the endogenous mRNA exhibits a median intracellular half‐life of 9 h,^[^ ^61^ ^]^ whereas exogenous mRNA undergoes more rapid degradation within a time frame of 1–4 h.^[^ ^62^ ^]^ Meanwhile, the formation of CPP/mRNA complexes can enhance intracellular stability and prolong protein expression (**Figure** **4**A).^[^ ^63^ ^]^ Moreover, CPPs are highly suitable for peptide‐based carriers due to their facile design and synthesis through solid‐phase peptide synthesis.

Protamine, a naturally occurring protein rich in L‐arginine, has the capacity to complex nucleic acids (DNA and RNA) and protect them from enzymatic degradation within biological systems.^[^ ^64^ ^]^ CureVac has established the RNActive platform, which is based on protamine/mRNA complexes, with pipeline products including CV9103, CV9104,^[^ ^65^ ^]^ and CV9202 that have advanced to various clinical stages for tumors.^[^ ^66^ ^]^ Fan et al. recently developed SmartNeo, a computational tool for predicting immunogenic neoantigens. This tool linearly arranges identified candidates and encapsulates mRNA encoding 20 prioritized neoantigen peptides within a lipopolyplex (LPP) with a protamine/mRNA core and lipid‐shell structure. The therapeutic efficacy of this approach was validated in CT26, MC38, and B16F10 murine tumor models (Figure 4B).^[^ ^67^ ^]^

#### 3.1.2 Lipid‐Based Delivery Systems

Liposomes are spherical vesicles composed of one or more phospholipid layers that resemble the structure of cell membranes. Liposomes are effective carriers for delivering mRNA with low toxicity and high biocompatibility. The exploration of liposomes as vehicles for mRNA delivery dates back to the 1960s.^[^ ^68^ ^]^ However, early liposomes encountered limited delivery efficiency and inadequate stability. LNP has been developed to address these issues, and it is widely recognized as one of the most advanced and extensively utilized platforms for mRNA delivery (Figure 4C). The classic formulation typically comprises ionized lipids, cholesterol, auxiliary lipids, and PEGylated lipids. These components could enhance loading efficiency, facilitate efficient delivery, and minimize toxicity.

#### 3.1.3 Polymeric Delivery Systems

Polymeric nanoparticles (PNP), distinguished by their structural diversity, ease of functionalization, and remarkable stability, are pivotal for the efficient delivery of mRNA. These systems encompass polymeric micelles, polymeric vesicles, and hybrid nanoparticles.

Cationic polymer nanoparticles (Figure 4D), formed from block copolymers, possess cationic segments that are electrostatically complex with mRNA, thereby protecting the mRNA from enzymatic degradation. These polymers include polyethyleneimine (PEI), polyesters, poly amino acids, and dendrimers. Among these, PEI stands out as the most extensively studied and commercially viable cationic polymer.^[^ ^69^ ^]^ For example, by combining the hydrogel with mRNA/PEI, a synergistic effect can be achieved, resulting in efficient encapsulation and robust protection of the mRNA. Yin et al. reported the development of a hydrogel formed from graphene oxide (GO) and PEI, capable of producing vaccines loaded with mRNA (ovalbumin, a representative antigen) and adjuvants (R848). The mRNA vaccine remained active in the body for 30 days, effectively preventing tumor growth and metastasis.^[^ ^70^ ^]^

Polymeric vesicles are formed through the self‐assembly of amphiphilic block copolymers. For example, the single‐component multifunctional sequence‐defined ionizable amphiphilic Janus dendrimer (IAJD) is a synthetic delivery system that has been demonstrated to enable efficient mRNA delivery. It exhibits high potency even at low concentrations of ionizable amines. This dendrimer efficiently self‐assembles with mRNA into polymeric vesicles, exhibiting exceptional transfection efficiency both in vitro and in vivo.^[^ ^71^ ^]^

Lipid‐polymer hybrid nanoparticles (LPNs) effectively combine the beneficial properties of both liposomes and polymeric nanoparticles, capitalizing on the synergistic effects of these two delivery systems. There are two primary subtypes of LPN (Figure 4E). The first subtype is based on lipid nanoparticles augmented with helper polymers to enhance the properties of the formulations. The second subtype, named cationic lipid‐assisted nanoparticles (CLAN), is based on PEGylated helper polymers (amphiphilic block polymers) and utilizes cationic lipids (e.g., BHEM‐Chol, DOTAP) for enhanced mRNA encapsulation. These could be fabricated using single or double emulsification, resulting in distinct assembled structures. For instance, Fan et al. constructed a CLAN incorporating PLGA, PEG‐*b*‐PLGA, and BHEM‐Chol to efficiently deliver antigen‐encoding mRNA in cancer immunotherapy applications.^[^ ^72^ ^]^ Xu et al. utilized a similar CLAN to encapsulate Cas9 mRNA and gRNA‐targeted NLRP3, thereby improving the treatment of inflammatory diseases.^[^ ^73^ ^]^ The strategy was also applied to the other polymeric delivery systems, such as poly(β‐amino ester) (PBAE). A suite of PBAE has been designed and synthesized, demonstrating high transfection efficiency in the construction of mRNA delivery systems.^[^ ^74^ ^]^ For example, a team developed a lipid‐modified PBAE named L‐PBAE, which was further self‐assembled with PEG‐PLGA into a “particle‐in‐particle” nanostructure for enhanced delivery of pDNA and mRNA.^[^ ^75^ ^]^

### 3.2 Inorganic Delivery Systems

Inorganic nanoparticles have emerged as promising vehicles for mRNA delivery, as they can be engineered to have specific sizes, structures, and/or geometries, thereby facilitating various biological applications.^[^ ^76^ ^]^ In recent years, the use of inorganic nanoparticles for mRNA delivery has been extensively investigated, with studies exploring the applications of gold nanoparticles, silica nanoparticles, iron oxide nanoparticles, and metal ion‐polyphenol complexes.

#### 3.2.1 Gold Nanoparticles

Gold nanoparticles (AuNPs) possess excellent physicochemical properties, such as ease of surface functionalization, a high surface area‐to‐volume ratio, and low toxicity.^[^ ^77^ ^]^ Furthermore, AuNPs may enhance cellular uptake and transmembrane transport by optimizing particle size, shape, and surface charge. For example, AuNPs of different sizes were functionalized by Puntes and colleagues using 11‐amino‐1‐undecanethiol (AUT) or PEI, followed by mRNA adsorption, resulting in significantly improved stability and delivery efficiency in vivo.^[^ ^78^ ^]^ Additionally, AuNPs have been utilized to deliver gene‐editing tools, such as CRISPR‐Cas9 gRNA, opening new avenues for gene therapy.^[^ ^79^ ^]^

#### 3.2.2 Silica Nanoparticles

Mesoporous silica nanoparticles (MSNs), a type of silica nanoparticle, exhibit a honeycomb‐like structure with hollow channels, excellent biocompatibility, and tunability, making them one of the most commonly used silica‐based delivery carriers. They have garnered significant attention in the field of drug delivery. Due to the negatively charged surfaces of MSNs, various cationic macromolecules can be modified onto their surfaces. Building on this property, Dong et al. premixed mRNA with cationic polymers and bound it to the MSNs' surface electrostatically. They investigated how size, porosity, surface topography, and aspect ratio affect mRNA delivery, aiming to achieve efficient in vivo transfection without apparent toxicity.^[^ ^80^ ^]^ Wang et al. adopted an alternative strategy, which involved developing a silica nanocapsule conjugated with glucose and rabies virus glycoprotein peptides. This approach effectively bypassed the intact blood–brain barrier (BBB), enabling the delivery of CRISPR genome editors throughout the brain.^[^ ^81^ ^]^

#### 3.2.3 Iron Oxide Nanoparticles

Iron oxide nanoparticles mainly refer to those based on Fe₃O₄ or Fe₂O₃, which exhibit superparamagnetism at specific sizes. Iron oxide nanoparticles can electrostatically interact with anionic nucleic acids through cationization via surface engineering, making them excellent carriers for mRNA delivery. Huang et al. developed a nanoparticle with a thin charged layer of iron oxide at its core, an mRNA intermediate layer, and an outer layer composed of perfluorinated polyethyleneimine with heparin, capable of safely and efficiently delivering mRNA to tumor cells that are typically difficult to transfect.^[^ ^82^ ^]^ Iron oxide nanoparticles can also bind to lipid mixtures for the codelivery of mRNA. Grippin et al. developed a multifunctional mRNA‐nanoparticle platform with an iron oxide core and mixed lipids, which can be customized with tumor antigen mRNA to elicit a potent antitumor immune response, reprogram the tumor microenvironment in brain tumors and enable magnetic resonance imaging tracking of particle localization via the iron oxide core.^[^ ^83^ ^]^

#### 3.2.4 Metal Ion‐Polyphenol Complexes

**Figure 5:**
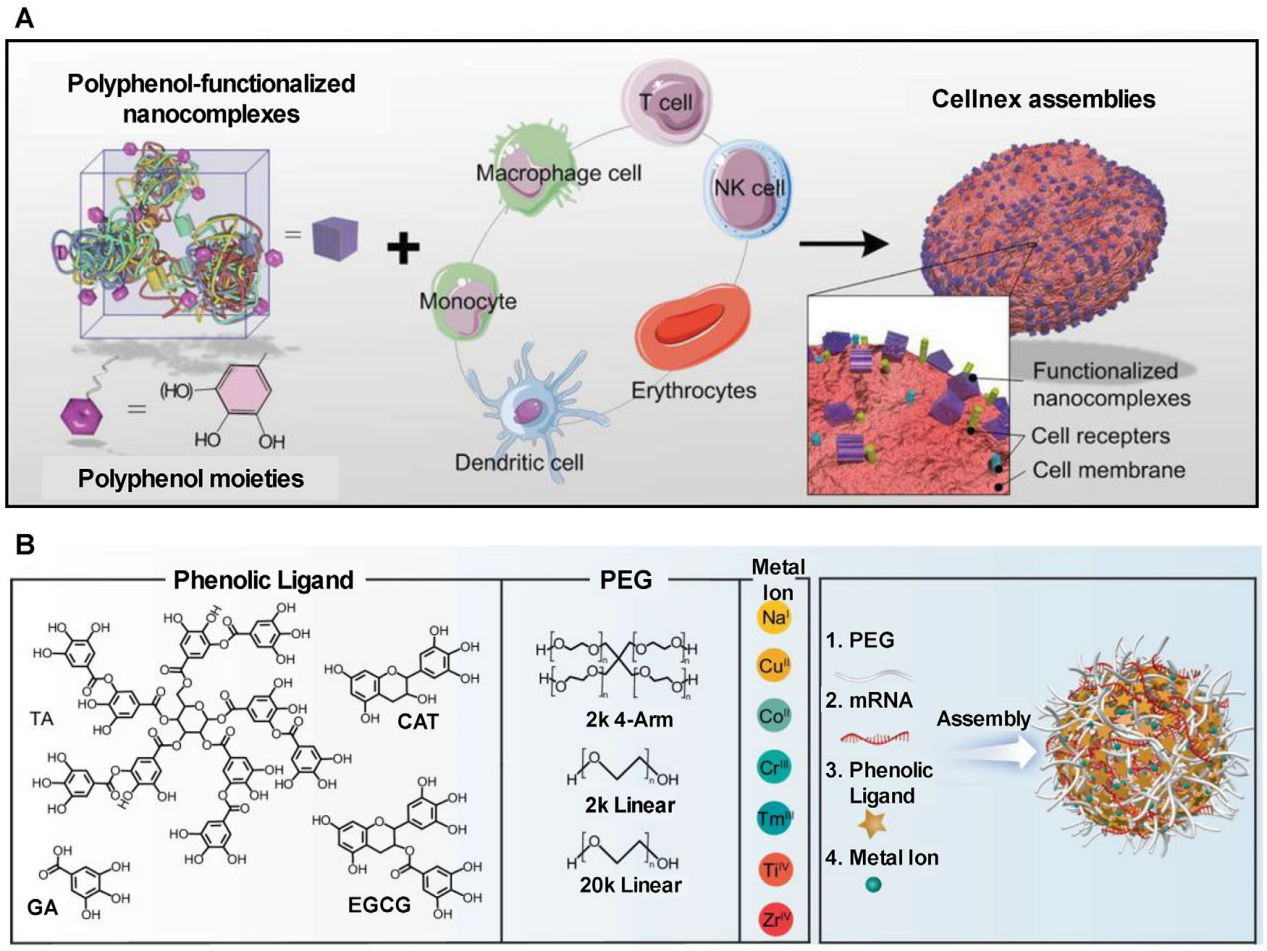
Metal ion‐polyphenol complexes. A) Scheme showing the preparation of Cellnex assemblies with Fe^3+^‐polyphenol functionalized nanocomplexes. Reproduced with permission.^[^ ^84^ ^]^ Copyright 2020, Wiley‐VCH. B) The diagram illustrating the formation of metal‐phenolic networks (mRNA‐MPN NPs) involving PEG, mRNA, phenolic ligand, and metal ion. Reproduced with permission.^[^ ^85^ ^]^ Copyright 2024, Spring Nature.

The employment of the metal ion‐polyphenol complexes has emerged as a novel approach for mRNA encapsulation and delivery in recent years. For example, Zhao et al. found that a specific combination of tannic acid and biomolecules, including nucleic acids, proteins, bioconjugators, and viral vectors, in precise proportions could produce nanocomplexes. And then, the Fe^3+^ ions could facilitate the aggregation of nanocomplexes on cellular surfaces by mitigating electrostatic repulsion forces for preparing Cellnex assemblies (**Figure** **5**A).^[^ ^84^ ^]^ Furthermore, Gu et al. developed noncationic and versatile metal‐phenolic networks, termed mRNA‐MPN NPs, in which mRNA was assembled into a PEG‐polyphenol network stabilized by metal ions. This innovative design enables efficient transfection in vivo and in vitro with high biocompatibility. Notably, the organ tropism of the nanoparticle can be readily modulated through modification of the composition and ratio of its building blocks (Figure 5B).^[^ ^85^ ^]^

### 3.3 Biomimetic Delivery Systems

Biomimetic delivery systems exhibit chemical, physical, or morphological similarities to biological structures. They demonstrate superior intelligent delivery capabilities in areas, such as barrier penetration, active targeting, and other relevant aspects. Exosomes and virus‐like particles (VLPs) are also the most commonly used biomimetic mRNA delivery systems.

#### 3.3.1 Extracellular Vesicles (EVs)

EVs, nonreplicating particles secreted by cells and enclosed by a lipid bilayer, are categorized into ectosomes and exosomes. The latter has received substantial research focus, is in the 40–150 nm size range, and expresses CD9, CD63, and CD81 markers.^[^ ^86^ ^]^ Their excellent biocompatibility and low immunogenicity make them ideal candidates for delivering mRNA‐based drugs. Moreover, exosomes can acquire enhanced specific cell targeting capability and barrier penetration ability through appropriate modification. Xiao et al. designed an exosome delivery platform that expresses anti‐CD3/CD28 scFvs, thereby loading CAR mRNA to produce CAR T cells with the capacity to kill cancer cells in vitro.^[^ ^36^ ^]^ However, incorporating mRNAs into exosomes yields low results. Yang et al. have developed a cellular nanoporation technique that produces up to 50‐fold more exosomes and achieves a more than 1000‐fold increase in therapeutic mRNAs. Additionally, the exosomes containing PTEN mRNA enhanced the inhibition of glioblastoma multiforme.^[^ ^87^ ^]^

#### 3.3.2 Virus‐Like Particles (VLPs)

**Figure 6:**
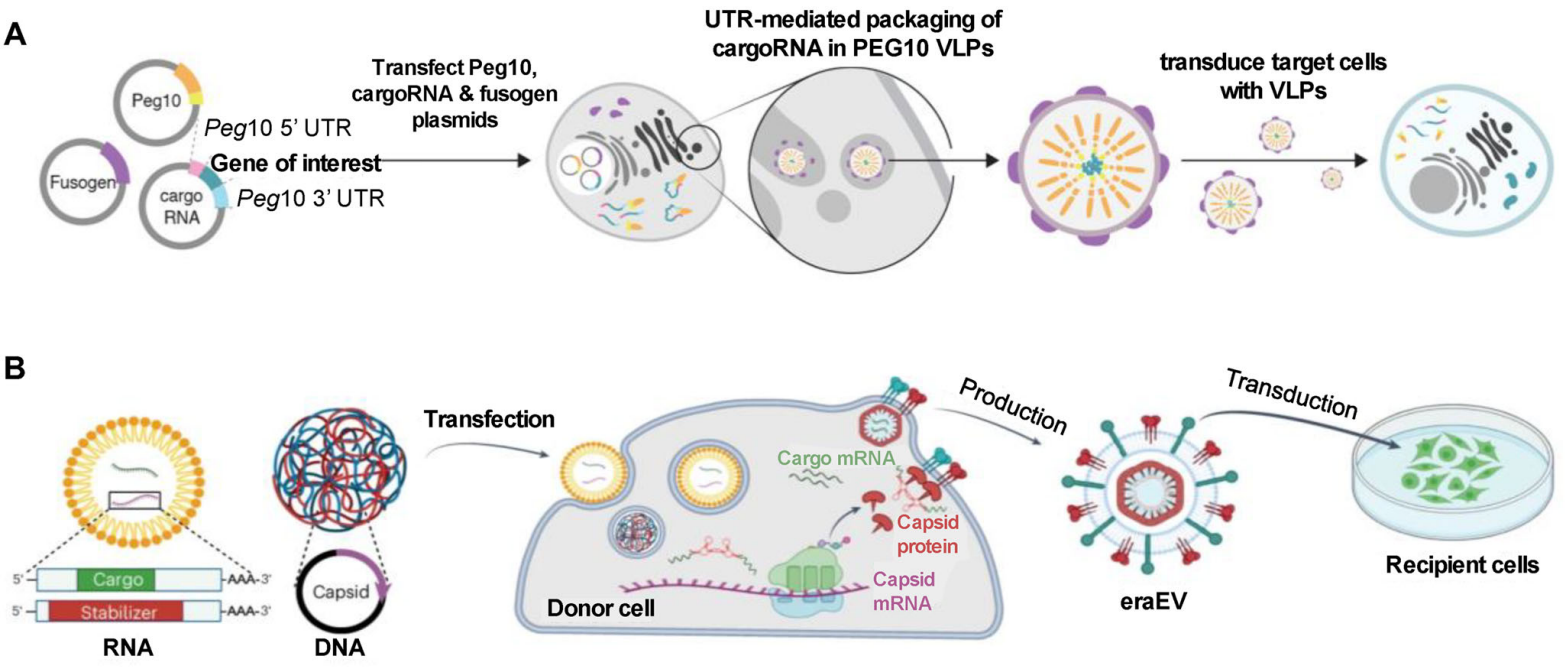
Virus‐like particles (VLPs). A) Selective Endogenous eNcapsidation for cellular Delivery (SEND) was engineered for genome engineering. Reproduced with permission.^[^ ^89^ ^]^ Copyright 2021, AAAS. B) Illustration of the workflow for transfecting donor cells to produce extracellular vesicles (EVs) genetically modified with the activity‐regulated cytoskeleton‐associated (Arc) protein, enabling cargo mRNA loading and transfection. Reproduced with permission.^[^ ^90^ ^]^ Copyright 2024, Springer Nature.

VLPs are particles formed by the self‐assembly of viral structural proteins that mimic the natural structure of viruses but contain nonviral materials. Thus, VLPs replicate the efficient delivery capabilities of natural viruses while maintaining biosafety, making them an essential tool for mRNA delivery.^[^ ^88^ ^]^ PEG10, a mammalian retrovirus‐like protein, preferentially binds and secretes its mRNA in extracellular virus‐like capsids, and the cargo can be reprogrammed by flanking genes of interest with its untranslated regions. Segel et al. developed a novel RNA delivery platform called Selective Endogenous eNcapsidation for Cellular Delivery (SEND), which utilizes engineered PEG10 to package, secrete, and deliver specific RNAs. This platform successfully delivered the CRISPR‐Cas9 gene editing system to mouse and human cells (**Figure** **6**A).^[^ ^89^ ^]^ Additionally, Gu et al. introduced DNA or RNA encoding the capsid‐forming activity‐regulated cytoskeleton‐associated (Arc) protein and capsid‐stabilizing Arc 5'‐untranslated‐region RNA elements into immortalized and primary bone marrow‐derived leukocytes. The engineered EVs acquire endothelial adhesion molecules from donor leukocytes, attract endogenous enveloping proteins, cross the blood–brain barrier, and enter neurons at neuro‐inflammatory sites (Figure 6B).^[^ ^90^ ^]^

#### 3.3.3 Biomolecular Condensates

Research has revealed that biomolecules within cells, including millions of protein molecules and other biopolymers, are not uniformly distributed. Instead, they form membrane‐less, droplet‐like compartments through liquid–liquid phase separation (LLPS), referred to as condensates or coacervates. These coacervates exhibit remarkable capabilities in selectively recruiting, storing, and releasing macromolecules, including proteins, peptides, and nucleic acids. Their inherent dynamic properties endow them with exceptional potential for direct cytosolic delivery, positioning them as emerging platforms for nucleic acid‐based therapeutics and drug delivery systems.^[^ ^91^ ^]^

**Figure 7:**
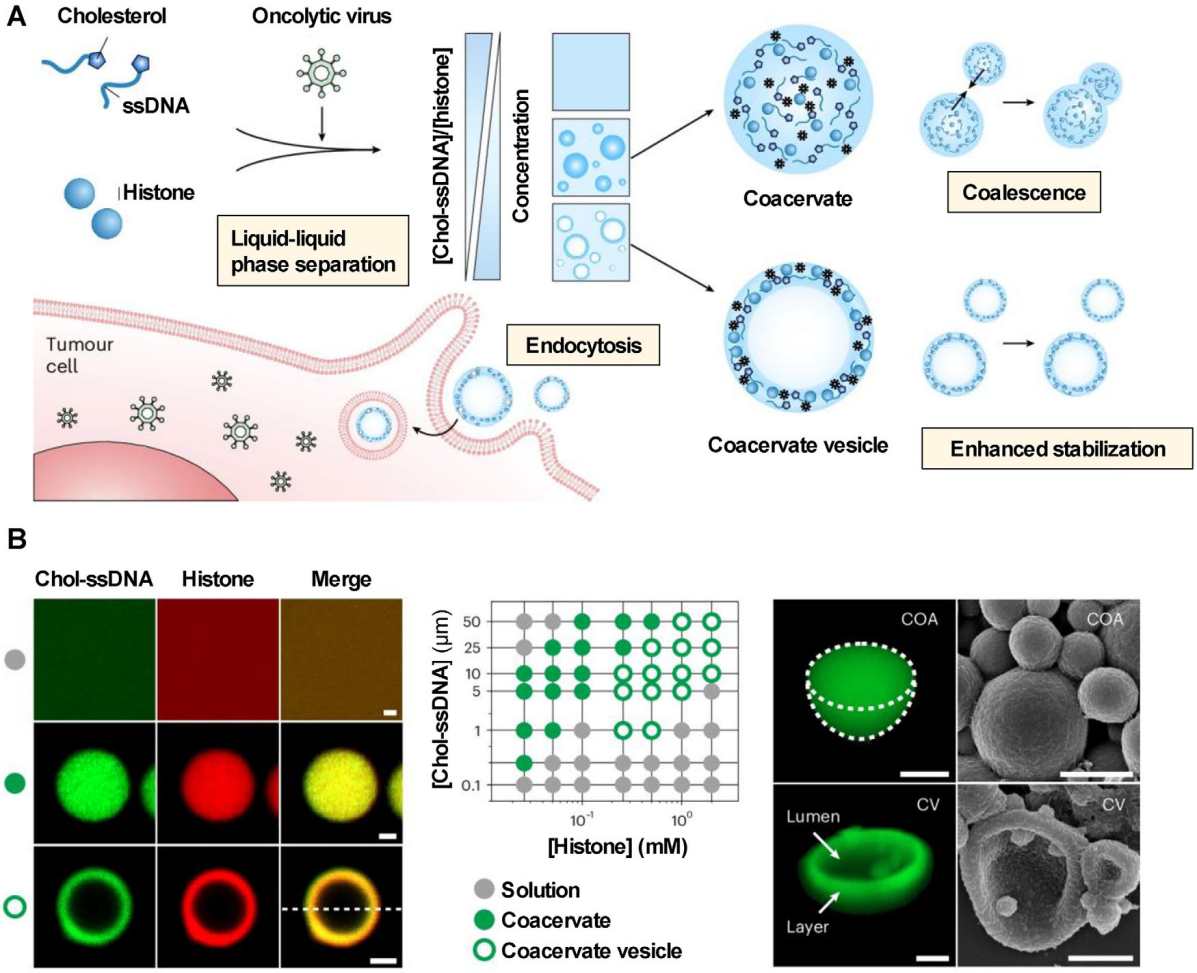
A) Schematic illustration of coacervate vesicle (CV) assembly and drug delivery process. B) Characterization of CV. Cholesterol‐modified single‐strand DNA (Chol‐ssDNA) forms a dense liquid layer around aqueous compartments, accompanied by histones at specific concentrations, through liquid–liquid phase separation (LLPS). Reproduced with permission.^[^ ^94^ ^]^ Copyright 2025, Springer Nature.

Notably, phase‐separating peptides (PSPs) self‐assembling into condensate microdroplets represent a promising class of intracellular delivery vehicles. Sun et al. reported the development of conjugated peptides capable of forming pH‐ and redox‐responsive coacervate microdroplets through LLPS. These microdroplets rapidly concentrate macromolecules, including mRNA, and facilitate direct cytoplasmic delivery, followed by glutathione‐triggered release of the payload, resulting in high mRNA transfection efficiency.^[^ ^92^ ^]^ Subsequent work by the same group systematically engineered PSPs with varied sequences, elucidating structure–function relationships between peptide architecture, condensate formation, and macromolecular delivery efficacy. Optimized PSPs demonstrated superior gene delivery performance in fibroblasts and immune cells.^[^ ^93^ ^]^ Inspired by natural vesicular compartments, Wen et al. recently described a novel LLPS‐driven vesicular assembly based on nucleic acid‐protein complexes. This system exploits the composition‐dependent self‐assembly of cholesterol‐modified single‐strand DNA (Chol‐ssDNA) with histones. Unlike classical coacervate, the resulting coacervate vesicles (CV) exhibit a unique architecture featuring aqueous spaces enclosed by high‐density boundary layers. The CV demonstrate enhanced capabilities in concentrating therapeutic cargo, such as oncolytic viruses and mRNA. Notably, they achieve a 48‐fold internalization efficiency in cancer cells through receptor‐independent and energy‐dependent macropinocytosis pathways (**Figure** **7**).^[^ ^94^ ^]^ Notably, researchers at Westlake University have identified a mammalian endogenous protein (PCT/CN2024/099339) that can self‐assemble with nucleic acids into coacervates through LLPS. The coacervates enable efficient mRNA transfection in primary T cells, natural killer (NK) cells, and hematopoietic stem cells, with minimal cytotoxicity. This innovative technology has been applied to engineer mRNA‐based CAR‐T cells.^[^ ^95^ ^]^

## 4 Precise mRNA Delivery Systems

Despite substantial progress in mRNA therapies during preclinical research, the clinical translation of mRNA nanomedicine remains fraught with challenges in the postpandemic landscape. In this context, it is imperative to reiterate that achieving the precision of mRNA therapeutics remains a formidable challenge in bridging the gap from the laboratory bench to the clinical bedside. In oncology, the success of mRNA therapy hinges significantly on the targeted delivery of mRNA therapeutics, a function primarily governed by the delivery systems. An ideal targeted delivery system should enhance the enrichment of mRNA in the intended organ and cell types, thereby reducing its accumulation in nontarget tissues. To achieve this precision, several key strategies are employed. 1) Enhancing the organ or cell targeting of the delivery system through passive or active targeting strategies to augment the specificity and efficacy of mRNA delivery. 2) Developing stimulus‐responsive delivery systems that can achieve controlled release of mRNA under endogenous or exogenous stimuli, thereby enabling precise temporal and spatial control over gene expression; 3) Exploiting the advantages of various drug delivery routes to optimize therapeutic efficacy while minimizing systemic side effects.

### 4.1 Targeting Strategies

#### 4.1.1 Passive Targeting

This approach is predicated on the distinct physicochemical characteristics inherent to the carrier. Commercial LNP has been documented to exhibit a propensity for hepatotropism, which complicates the assurance of mRNA therapeutics' efficacy on extrahepatic tumors.^[^ ^96^ ^]^

**Figure 8:**
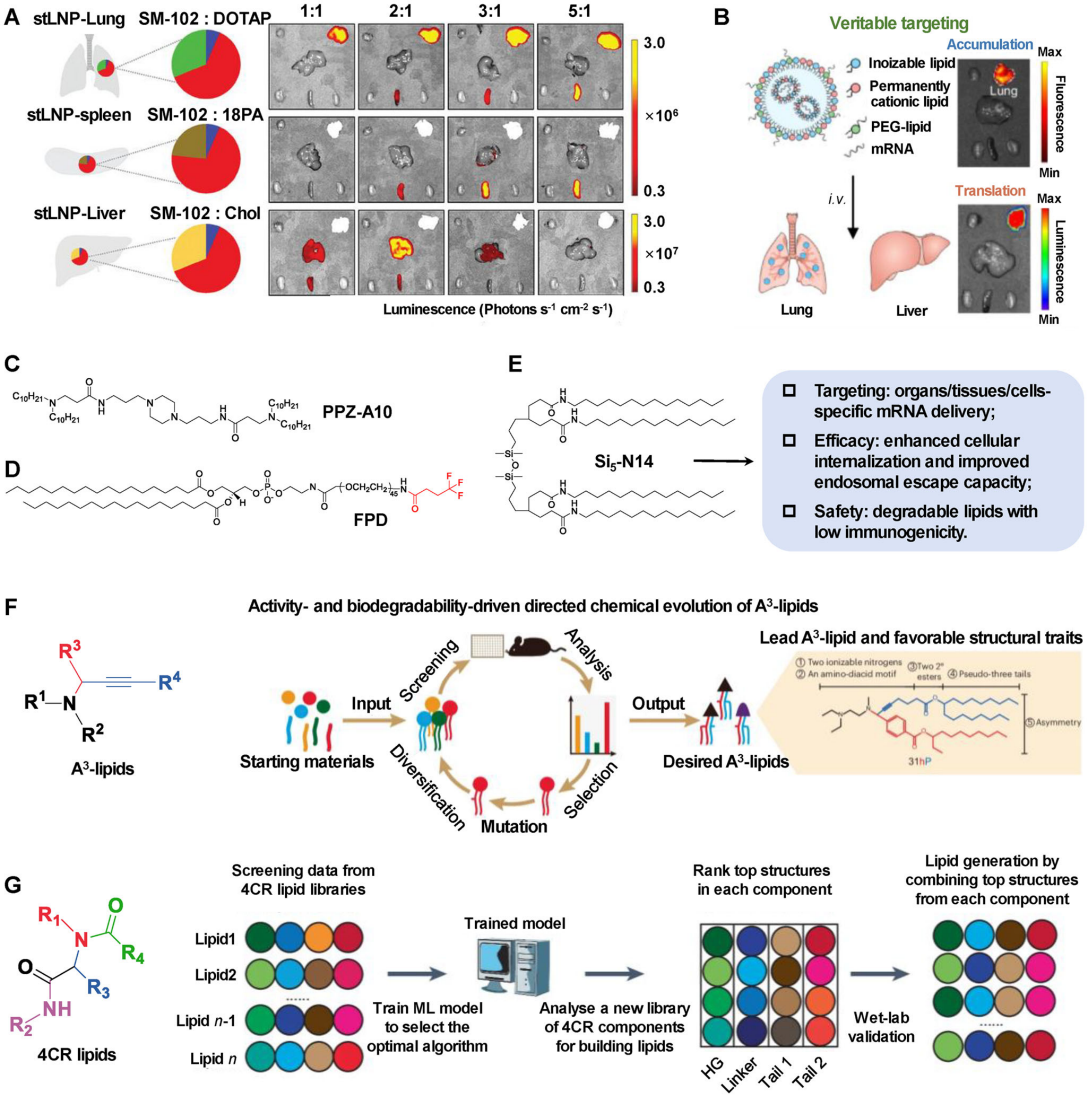
A) The 3‐component LNP (simplified targeted LNP, stLNP) enables lungs, spleen, and liver‐specific mRNA expression; Reproduced with permission.^[^ ^99^ ^]^ Copyright 2024, Wiley‐VCH. B) Representative mRNA accumulation and translation images from 6Ac1‐C12 3‐Comp Lung LNP. Reproduced with permission.^[^ ^100^ ^]^ Copyright 2024, Springer Nature. C) Piperazine‐derived lipid. Reproduced with permission.^[^ ^102^ ^]^ Copyright 2022, Springer Nature. D) Amidine‐incorporated degradable lipids. Reproduced with permission.^[^ ^103^ ^]^ Copyright 2024, American Chemical Society. E) Siloxane‐incorporated lipids could improve the targeting, efficacy, and safety of LNP. Reproduced with permission.^[^ ^104^ ^]^ Copyright 2025, Springer Nature. F) Directed chemical evolution could accelerate the structural optimization of propargylamine‐based ionizable lipids (A3‐lipids). Reproduced with permission.^[^ ^107^ ^]^ Copyright 2024, Springer Nature. G) Machine learning combined with combinatorial chemistry provides a synergistic approach to accelerate the discovery of ionizable lipids through a four‐component (4CR)‐enabled high‐throughput synthesis platform. Reproduced with permission.^[^ ^108^ ^]^ Copyright 2024, Springer Nature.

Modifying the formulation to control their size, zeta potential, morphology, protein corona, and other attributes to achieve organ‐selective targeting is a promising strategy. For example, the SORT (Selective Organ Targeting) system introduces a fifth component to the standard four‐component LNP formulation, thereby enabling precise targeting of the lungs, liver, and spleen.^[^ ^97^ ^]^ Moreover, Lian et al. have identified that incorporating specific covalent lipids or cross‐linkers into a four‐component LNP formulation can guide bone marrow delivery tropism, which is beneficial for treating hematopoietic disorders. The delivery efficiency is likely correlated with the structure and functionality of the integrated molecules.^[^ ^98^ ^]^ Notably, Fei et al. have devised an integrated strategy known as SELECT (Simplified LNP with Engineered mRNA for selective Cell‐type Targeting), which facilitates the targeted delivery of mRNA to the lungs, liver, and spleen through a three‐component LNP platform (**Figure** **8**A).^[^ ^99^ ^]^ Su et al. have suggested that cholesterol and phospholipids in the conventional four‐component LNP are dispensable. To simplify the LNP and enhance organ‐targeting capabilities, they have engineered a degradable‐core‐based ionizable lipid library and developed a 3‐component LNP composed of an ionizable lipid, a permanently cationic lipid, and a PEG‐lipid. The 6Ac1‐C12 3‐Comp Lung LNP has been shown to facilitate lung‐targeted mRNA accumulation and translation (Figure 8B).^[^ ^100^ ^]^

Furthermore, novel lipids have significantly augmented the targeting proficiency of LNP. Han et al. synthesized and screened imidazole‐incorporated degradable lipids through a designed amine‐thiol‐acrylate conjugation based on a one‐pot multicomponent reaction. By examining the structure–activity relationship of these lipids, they have identified a tail‐like amine‐ring‐alkyl aniline amine (12T‐O14) that selectively redirects LNP to the lungs or spleen.^[^ ^101^ ^]^ Building upon piperazine's established role in bioactive compounds, Ni et al. rationally engineered ionizable lipids (Pi‐Lipids) featuring a piperazine core flanked by two tertiary amines and hydrophobic R‐tail groups. Utilizing high‐throughput DNA‐barcoding technology, they systematically quantified the cell‐type‐specific targeting capabilities of 65 distinct Pi‐Lipid nanoparticles (Pi‐LNP). PPZ‐A10‐containing Pi‐LNP demonstrated superior delivery efficiency to immune cells compared to benchmark formulations (Figure 8C).^[^ ^102^ ^]^ Zhang et al. reported the development of fluorinated 1,2‐distearoyl‐sn‐glycero‐3‐phosphoethanolamine‐poly(ethylene glycol)‐2000 (PEG‐DSPE), designated FPD. Mechanistic studies revealed that FPD potentiated LNP cellular internalization while enhancing endosomal escape capacity, resulting in significantly improved mRNA transfection efficiency in B16F10 tumor cells and primary dendritic cells (Figure 8D).^[^ ^103^ ^]^ Xue et al. engineered a class of structurally diversified siloxane‐based ionizable lipids. They formulated siloxane‐incorporated LNP to control the organ‐specific delivery of mRNA to the liver, lungs, and spleen (Figure 8E).^[^ ^104^ ^]^

However, this process often necessitates extensive material or formulation screening. Cutting‐edge technology can rationally streamline the process. For instance, incorporating barcodes into nanoparticles enables tracking there in vivo fate.^[^ ^105^ ^]^ Rhym et al. leveraged peptide‐encoded mRNA barcodes for in vivo high‐throughput screening of LNP, employing liquid chromatography and tandem mass spectrometry to detect translated peptides. This approach evaluated over 400 nanoparticle formulations and 384 unique ionizable lipids in just nine mice.^[^ ^106^ ^]^ Additionally, advanced chemical strategy. Han et al. established a rational design strategy integrating iterative chemical derivatization with combinatorial chemistry, employing amine‐aldehyde‐alkyne coupling reactions as a modular framework. This approach enables accelerated discovery of propargylamine‐based ionizable lipids (A3‐lipids), achieving systematic optimization of mRNA delivery potency and biodegradability (Figure 8F).^[^ ^107^ ^]^ Then, applying artificial intelligence and other advanced technologies can expedite the optimization and design of delivery vehicles or nucleic acids; however, establishing a comprehensive large database is a crucial prerequisite for computational analysis and optimization. For example, Li et al. introduced a new high‐throughput screening platform based on a four‐component reaction to generate new ionizable lipids. And machine learning and combinatorial chemistry were also utilized to accelerate the process. They established a foundational dataset comprising 584 ionizable lipids and applied it to screen a comprehensive virtual library of 40 000 lipids, ultimately identifying that 119–23 surpassed the benchmark lipid (Figure 8G).^[^ ^108^ ^]^ Furthermore, Xu et al. developed the AI‐guided Ionizable Lipid Engineering (AGILE) platform, which integrates deep learning with high‐throughput combinatorial lipid synthesis to elucidate the complex relationship between the molecular structure of ionizable lipids and their mRNA transfection efficiency.^[^ ^109^ ^]^

#### 4.1.2 Active Targeting

Modifying nanoparticles with active targeting strategies is a direct approach to achieving substantial enrichment of target cells. For example, most work on generating CAR T cells in vivo using nanoparticles has utilized targeted ligand‐mediated T cell activation, such as CD3^[^ ^38^ ^]^ and CD8.^[^ ^110^ ^]^ Examples of such ligands also include hyaluronic acid,^[^ ^111^ ^]^ folic acid,^[^ ^112^ ^]^ phenylboronic acid,^[^ ^113^ ^]^ proteins (transferrin,^[^ ^114^ ^]^ gelatin,^[^ ^115^ ^]^ monoclonal antibodies^[^ ^51^ ^]^), peptides,^[^ ^116^ ^]^ and aptamers.^[^ ^117^ ^]^ Based on the mode of combination, these interactions can be categorized into two main types: covalent conjugation mediated by click chemistry and biorthogonal chemistry, and noncovalent interactions, which include electrostatic interactions, hydrophobic interactions, and hydrogen bonds.

**Figure 9:**
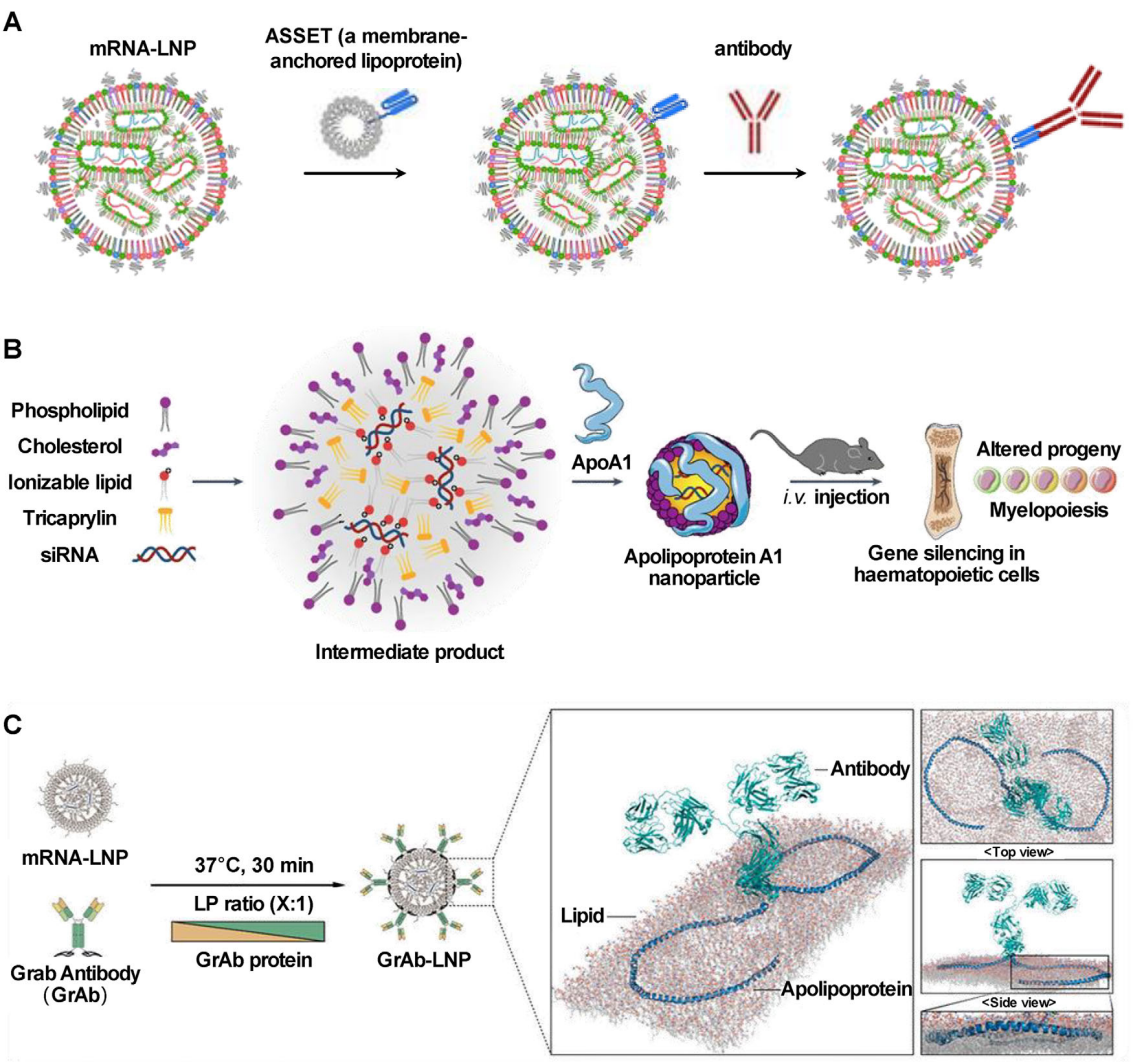
Lipoprotein‐based postmodification strategy for active targeting. A) Schematic of ASSET (anchored secondary scFv enabling targeting)‐coated LNP preparation for binding targeting antibodies. Reproduced with permission.^[^ ^51^ ^]^ Copyright 2018, Springer Nature. Reproduced with permission.^[^ ^120^ ^]^ Copyright 2020, AAAS. B) Scheme of Apolipoprotein A1 LNP (with PEGylated lipid replaced by tricaprylin) for delivering RNA to myeloid cells and their bone marrow progenitors. Reproduced with permission.^[^ ^121^ ^]^ Copyright 2025, Springer Nature. C) GrAb antibody anchors mRNA‐LNP via apolipoprotein's high natural lipid‐binding affinity, presenting antibodies for active targeting. Reproduced with permission.^[^ ^122^ ^]^ Copyright 2025, American Chemical Society.

Two surface modification strategies have been developed for active targeting of LNP. 1) Premodification strategy. Here, ligand molecules are preattached to lipid molecules, which subsequently self‐assemble with other components to form LNP or the ligands are incorporated into the LNP. For instance, phosphatidylserine was incorporated into LNP, facilitating recognition and binding by macrophage surface receptors and thereby enhancing selective delivery.^[^ ^41^ ^]^ DSPE‐PEG2K‐Biotin could be incorporated into LNP to facilitate the streptavidin‐biotin system for attaching anti‐CD3 antibodies.^[^ ^118^ ^]^ 2) Postmodification strategy. While click chemistry remains a cornerstone for covalent nanoparticle surface engineering,^[^ ^119^ ^]^ emerging biological paradigms exploit endogenous lipoprotein‐lipid interactions to achieve precision modifications. Pioneering this approach, Peer et al. developed ASSET (Anchored Secondary scFv Enabling Targeting)—a biomimetic platform comprising a membrane‐anchored lipoprotein fused to an antirat IgG2a scFv. The ASSET‐coated LNP utilizes high‐affinity scFv‐Fc interactions with rat IgG2a to facilitate antibody‐directed cell targeting, achieving success in nucleic acid delivery and gene editing (**Figure** **9**A).^[^ ^51^, ^120^ ^]^ Moreover, Hofstraat and colleagues developed a nanoparticle platform based on apolipoprotein A1 (apoA1), which could be readily modified through simple mixing. They demonstrated that the apoA1‐coated LNP could deliver RNA to myeloid cells and their bone marrow progenitors (Figure 9B).^[^ ^121^ ^]^ Furthermore, Park et al. engineered antibodies fused with apolipoproteins (GrAbs). Leveraging the natural lipid‐binding affinity of apolipoproteins, they achieved spontaneous functionalization of GrAbs on the surface of LNP. The team further constructed LNP containing GrAbs targeting HER2 and encapsulating p53 mRNA, allowing tumor targeting and growth inhibition (Figure 9C).^[^ ^122^ ^]^

Despite the promise of active targeting strategies, several challenges should be addressed. 1) Monoclonal antibody challenges. Despite their targeted binding to cellular receptors, monoclonal antibodies are limited by inefficient chemical conjugation processes. This necessitates individual optimization for each antibody, complicating quality control. 2) Receptor protein constraints. The distribution and abundance of receptor proteins can limit the efficacy of targeted therapies. 3) Cancer heterogeneity. The diversity of cancer cells can render single receptor targeting ineffective, and losing one receptor can lead to drug resistance. 4) Protein corona effect. The in vivo formation of a protein corona around nanoparticles can impede the specific binding of targeted molecules, potentially undermining therapeutic specificity and efficacy.

### 4.2 Stimuli‐Responsive Delivery Systems

To realize the potential of mRNA therapy, developing sophisticated delivery systems is essential for the precise and efficient delivery of mRNA to tumor tissue or its release into the cytoplasm. The evolution of nanoparticles has led to the development of drug‐delivery systems that have transitioned from passive carriers to stimuli‐responsive platforms. Thus, the next frontier in the delivery system is expected to involve the controlled release of mRNA in response to internal and external stimuli. For instance, delivery systems can be activated by the tumor microenvironment (e.g., pH, glutathione, reactive oxygen species, enzymes) or exogenous stimuli (e.g., light, sound, electricity, heat, magnetism), enabling the controlled delivery and release of mRNA.^[^ ^123^ ^]^ This section provides a concise overview of mRNA delivery systems that respond to pH and ultrasound stimuli.

#### 4.2.1 mRNA Delivery Systems Response to pH

The tumor microenvironment is generally considered acidic, with an average pH value of 6.8 across various cancer types.^[^ ^124^ ^]^ After the cell's internalization, the particles are transported along various organelles, from an early endosome pH range of ≈6.1–6.8 to a late endosome pH range of 4.8–5.6, ultimately reaching a final lysosome pH of 4.5.^[^ ^125^ ^]^ Based on this, pH‐sensitive groups are used to design mRNA delivery vehicles or lipid materials to ensure that the nanoparticles are stable under normal physiological conditions (pH 7.0–7.4) but respond to changes in acidic environments, leading to changes or disruption in the physicochemical properties of the carrier or material to promote cellular internalization of the carrier or endosomal escape of mRNA. The general structures are as follows.

**Figure 10:**
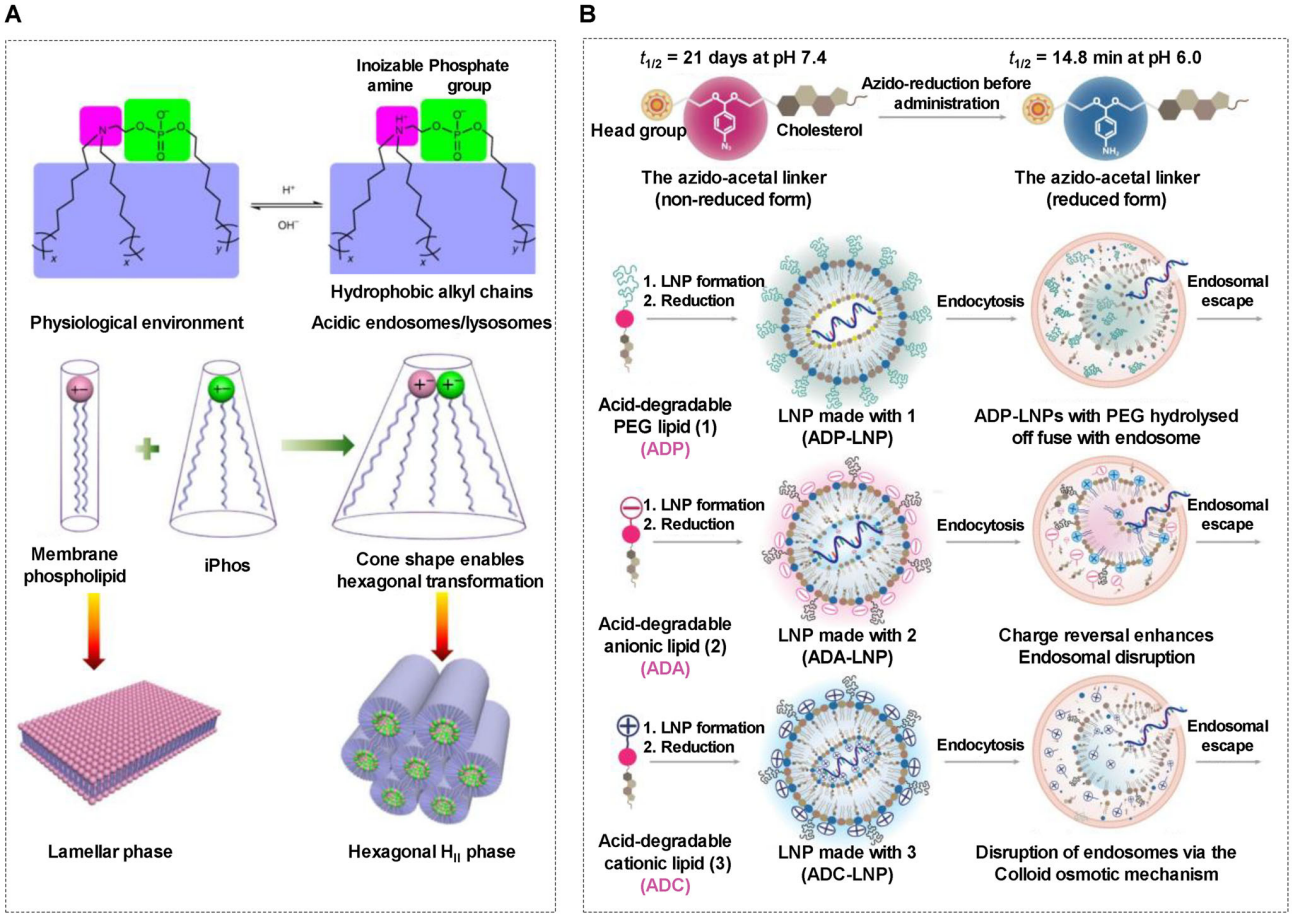
mRNA delivery systems respond to pH. A) Typical acid‐protonation structures. Multitailed ionizable phospholipids (iPhos) facilitated membrane insertion and enhanced endosomal escape through a hexagonal phase transition. Reproduced with permission.^[^ ^127^ ^]^ Copyright 2021, Springer Nature. B) Typical acid‐degradable structures. The “azido‐acetal” linker was stable at pH 7.4 (nonreduced form) but rapidly hydrolyzed in endosomes (reduced form), with a hydrolysis half‐life (t_½_) of 14.8 min at pH 6.0. LNP made with the acid‐degradable lipid‐modified with different head groups facilitated endosomal escape via various mechanisms. Reproduced with permission.^[^ ^132^ ^]^ Copyright 2024, Springer Nature.

1) Acid‐protonation structures. Tertiary amines are the most commonly used structures. For example, ionizable lipids in LNP (with an optimal p*K*a considered to be 6.2–6.5)^[^ ^126^ ^]^ are designed to enhance the endosomal escape of nanoparticles to achieve cytoplasmic delivery of mRNA. For instance, Liu et al. reported the multitailed ionizable phospholipids (iPhos), the protonation of the tertiary amine facilitated membrane hexagonal transformation, and subsequent cargo release from endosomes (**Figure** **10**A).^[^ ^127^ ^]^ In another case, poly(diisopropylaminoethyl methacrylate) (PDPA), Poly (2‐Hydroxyethyl Methacrylate) (PHMEMA), and PBAE have a relatively high concentration of ionizable tertiary amines in their backbone or side chains, which can provide buffering in the endosomal compartment and promote endosomal escape.^[^ ^128^ ^]^ Additionally, cytosine‐rich i‐motifs have been used in drug delivery systems and respond to acidic conditions (pH 4.0–6.0).^[^ ^129^ ^]^ Fu et al. developed a DNA gel for mRNA delivery, utilizing pH‐responsive i‐motif structures to achieve endosomal escape of mRNA. However, it is worth noting that lysosomes in normal tissue cells also have lower pH values and off‐target effects remain complex and cannot be entirely avoided at this stage.^[^ ^130^ ^]^

2) Acid‐degradable structures. Bonds such as benzylimidate, 2,3‐dimethylmaleic anhydride (DMAA), hydrazones, thioketals, vinyl ethers, orthoesters, ketals, acetals, and other bonds can degrade under specific pH conditions, thereby enhancing tumor cell uptake or facilitating the rapid release of mRNA. After cellular internalization, particles accumulate maximally in early endosomes within 40 min and then move to late endosome/lysosome, reaching a plateau after 100 min, indicating a limited time window for endosome escape.^[^ ^131^ ^]^ Consequently, Zhao et al. designed a rapidly degradable “azido‐acetal” acid‐degradable linker for preparing RD‐LNP with distinct head groups, which were successfully redirected to various organs and cells and escaped from endosomes (Figure 10B).^[^ ^132^ ^]^ Recent studies indicate that the tumor microenvironment is severely acidified (pH < 5.3), primarily due to lactate excretion by tumor cells, suggesting that pH‐responsive materials should reassess their design for cancer treatment.^[^ ^133^ ^]^

#### 4.2.2 mRNA Delivery Systems Response to Ultrasound

Focused ultrasound (FUS) concentrates sound waves within a precise region, typically 1–2 mm in diameter and 8–15 mm in depth, to achieve localized targeting and deep tissue penetration. Additionally, ultrasound‐targeted microbubble destruction (UTMD) represents an innovative approach to drug delivery, integrating FUS with intravascularly circulating microbubble (MB) contrast agents to elicit diverse biological responses that facilitate drug penetration. The principal mechanisms involve ultrasound‐induced cavitation, thermal effects, and sonoporation, which result in the transient opening of vascular endothelial tight junctions, tissue permeability, and cell membrane lysis, thereby augmenting the delivery of therapeutic agents into tumor tissues and enhancing intracellular uptake.^[^ ^134^ ^]^ This technique is primarily enabled by MB, which is filled with perfluorocarbon or other gases, endowing the delivery system with ultrasound responsiveness and contrast imaging capabilities.

**Figure 11:**
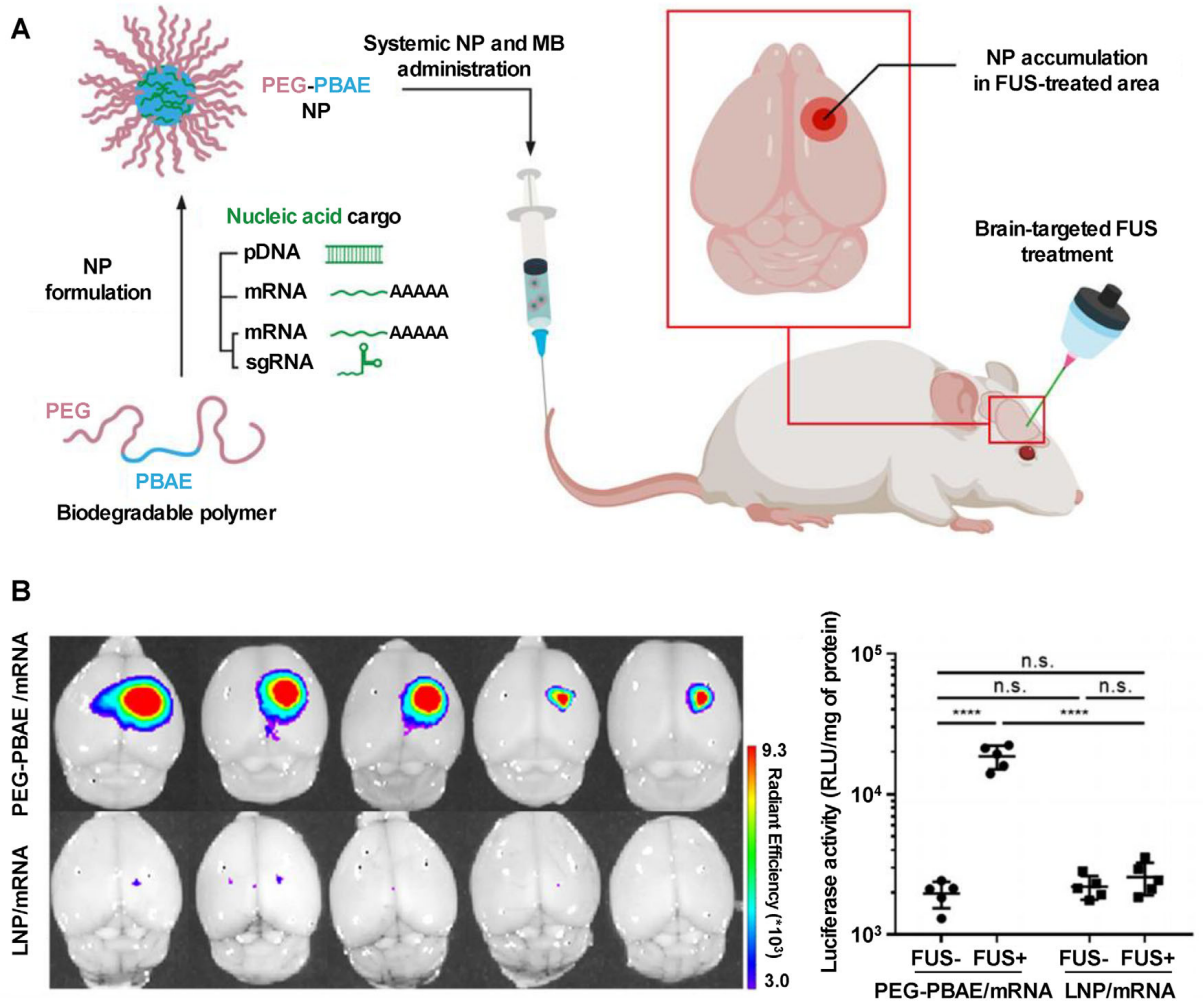
mRNA delivery systems response to ultrasound. A) PEG‐PBAE NP accumulated in the focused ultrasound (FUS) treated brain area following systemic nanoparticles and microbubbles (MB) administration for brain nucleic acid delivery, facilitated by FUS‐mediated blood–brain barrier (BBB) disruption. B) Systemic delivery of PEG‐PBAE/mRNA and FUS and MB treatment resulted in significant luciferase expression within the right striatum. Reproduced with permission.^[^ ^135^ ^]^ Copyright 2024, American Chemical Society.

UTMD has been applied in two primary ways. 1) Disrupting the BBB. For instance, Kwak et al. encapsulated various nucleic acids, including DNA, mRNA, and gRNA, within PEG‐PBAE polymer materials. They employed UTMD to breach the BBB, enabling localized nucleic acid delivery and gene editing in the brain (**Figure** **11**A,B).^[^ ^135^ ^]^ Similarly, Wang et al. integrated UTMD with anti‐PD‐L1 peptide DPPA‐modified LNP to selectively deliver IL‐15 mRNA to melanoma cells, which effectively suppressed tumor growth and recurrence.^[^ ^136^ ^]^ Ogawa et al. demonstrated the feasibility of using FUS to deliver mRNA‐LNP to the murine brain.^[^ ^137^ ^]^ 2) Facilitating direct cytoplasmic drug delivery. Lux's team modified the surface of MBs with maleimide to attach SpeDex/cGAMP and an anti‐CD11b antibody. By applying local FUS to the tumor, they successfully delivered cGAMP into the cytoplasm of APCs, enabling targeted tumor immunotherapy.^[^ ^138^ ^]^

### 4.3 Administration Routes

In contrast to parenteral administration methods like intravenous drug delivery, oral,^[^ ^139^ ^]^ inhaled,^[^ ^140^ ^]^ and transdermal administration^[^ ^141^ ^]^ offer unique benefits. Given the extensive coverage of this subject in existing literature,^[^ ^142^ ^]^ the subsequent section will delve into recent advancements in oral and pulmonary inhalation administration.

#### 4.3.1 Oral Administration

In reality, oral administration remains the most compliance‐friendly and anticipated drug delivery route. Oral mRNA is especially suitable for rapidly deploying intermittent interventions, such as vaccines, and is beneficial for long‐term therapeutic regimens.

However, the oral delivery of nucleic acids is impeded by several gastrointestinal barriers.^[^ ^143^ ^]^ 1) The acidic microenvironment with pH levels ranging from 1.0 to 7.5. 2). The threat of enzymatic degradation by endogenous nucleases. 3) The formidable mucus barrier, a complex hydrogel composed of water, mucins, glycan, etc., that separates the epithelial tissue from the lumen, with thicknesses reaching into the hundreds of micrometers. 4) The epithelial barrier, characterized by tight junctions linking epithelial cells, presents a substantial challenge for mRNA delivery.

Moreover, several innovative strategies and vectors have been developed to overcome these challenges. For instance, the inclusion of mRNA protectants such as β‐glucan, which form complexes with mRNA through hydrogen bonding, has been employed. These complexes exhibit responsive behavior to the varying pH environments along the gastrointestinal tract, partially dissociating under the acidic conditions of the stomach (pH ≈2.0) and reassociating in the more alkaline intestinal environment (pH ≥ 7.0) to protect the mRNA. Luo et al. have engineered an oral mRNA cancer vaccine by encapsulating β‐glucan/mRNA complexes within LNP using microfluidic technology. This encapsulation ensures the stability of complexes within the harsh gastrointestinal tract and their subsequent uptake by dendritic cells, activating the immune system.^[^ ^144^ ^]^

**Figure 12:**
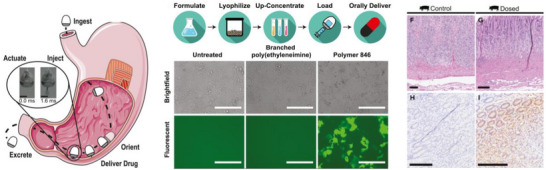
Oral mRNA delivery systems. Schematic illustrations depicting the mechanism, preparation, and mRNA transfection of Polymer 846/mRNA nanoparticles via a self‐orienting millimeter‐scale applicator (SOMA). Reproduced with permission.^[^ ^146^ ^]^ Copyright 2022, Cell Press.

Additionally, microscale capsules may serve as protective shells for mRNA delivery vectors, enriching the form of oral mRNA delivery. Traverso and colleagues previously developed the self‐orienting millimeter‐scale applicator (SOMA) to directly deliver drugs to the submucosal layer of the stomach, thereby bypassing degradative enzymes in the gastrointestinal tract.^[^ ^145^ ^]^ Furthermore, the team encapsulated the mRNA within branched hybrid PBAE nanoparticles (Polymer 846/mRNA NPs), which were then lyophilized and loaded into the SOMA. This approach facilitated gastric delivery and subsequent transfection upon dosing 150 µg of Cre mRNA in a porcine model (**Figure** **12**).^[^ ^146^ ^]^

#### 4.3.2 Pulmonary Administration

Pulmonary inhalation extends to nasal delivery, specifically the delivery of drugs to the lungs or lower respiratory tract. In the context of pulmonary mRNA therapy, the primary indications include protein replacement therapy, cancer vaccines, and cell therapy based on mRNA, with key targets, such as tumor cells, epithelial cells, and antigen‐presenting cells, among others. Actually, pulmonary inhalation is advantageous for several reasons, including 1) high patient compliance and ease of administration; 2) the ability to overcome the blood–air barrier, leading to direct pulmonary delivery without systemic drug exposure and associated high dosing costs; 3) safety, as it circumvents unnecessary brain drug exposure due to particle uptake in the upper respiratory tract; 4) high‐level pulmonary drug enrichment; 5) significant enhancement of drug absorption due to the sizeable alveolar surface area; 6) the ability to elicit potent mucosal immune responses via inhaled vaccination. Consequently, an ideal inhaled mRNA delivery system should be capable of achieving deep lung deposition and extended pulmonary retention.

**Figure 13:**
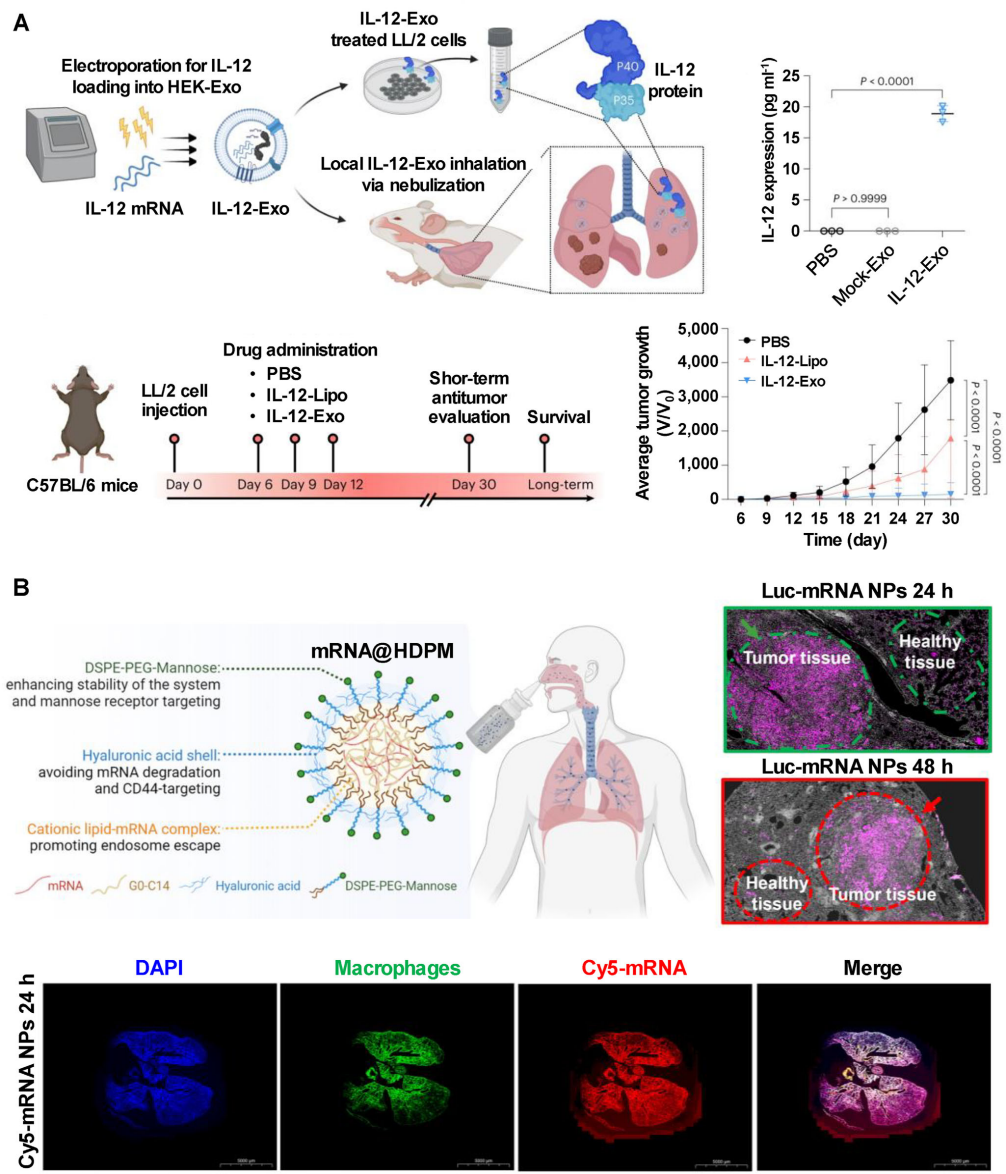
Pulmonary mRNA delivery systems. A) The schematic shows IL‐12 mRNA loaded into HEK‐Exo (IL‐12‐Exo), which is successfully expressed in the lungs of LL/2 tumor‐bearing mice and inhibits tumor growth. Reproduced with permission.^[^ ^150^ ^]^ Copyright 2024, Springer Nature. B) The scheme illustrates the key components and functions of mRNA@HDPM, which is found to accumulate effectively in lung tumor tissues and macrophages. Reproduced with permission.^[^ ^155^ ^]^ Copyright 2023, National Academy of Sciences.

Lung spheroid cells (LSC), a natural mixture comprising type I and II lung cells and mesenchymal cells, have been exploited by Cheng and colleagues.^[^ ^147^ ^]^ They demonstrated that exosomes derived from LSCs (LSC‐Exo) possess intrinsic phenotypes that enhance targeting and retention within the pulmonary environment.^[^ ^148^ ^]^ Wang et al. further improved the performance of LSC‐Exo by incorporating RBD‐PEG‐DSPE, which facilitates mucus penetration, prolongs retention in the respiratory tract and lung parenchyma, and promotes dendritic cell internalization of the exosomes.^[^ ^149^ ^]^ It is also an ideal candidate for mRNA inhaled delivery. For instance, Liu et al. investigated the therapeutic potential of exosome‐mediated delivery of mRNA‐encoding cytokines, such as IL‐12, for treating primary and metastatic lung tumors (**Figure** **13**A).^[^ ^150^ ^]^

As for other assembly delivery systems, the potent shear forces generated during nebulization may compromise the integrity of nanoparticle structures and induce aggregation.^[^ ^151^ ^]^ Consequently, a variety of specialized inhaled mRNA delivery systems have been developed. For example, the formulation and optimization of inhaled LNP has been extensively studied. The optimization of nebulized LNP primarily targets aspects, such as composition, ionizable lipid content, nebulization buffers, and excipients.^[^ ^152^ ^]^ Lokugamage et al. examined the formulation and design principles of nebulized LNP, highlighting that the molar concentration and ratio of PEG significantly influence the mRNA delivery efficiency of nebulized LNP. However, the precise mechanisms remain to be fully elucidated.^[^ ^153^ ^]^ In another study, Jiang et al. employed the design of experiments to refine the formulation of nebulized liposomal nanoparticles. They found that using sodium acetate (NaAc) as a nebulization buffer and incorporating 2% w/v branched poly(ethylene glycol) (bPEG20K) as an excipient enhanced the pulmonary mRNA delivery of nebulized lipoplexes. Their research also underscored the importance of ionizable lipids.^[^ ^151^ ^]^ Bai et al. optimized formulations and carefully selected buffers with diverse compositions, pH levels, alcohols, and nonionic surfactants to create LNP, demonstrating remarkable resistance to shear forces and potent mRNA expression.^[^ ^154^ ^]^ Furthermore, Tang et al. developed a delivery system based on cationic lipids and hyaluronic acid, functionalizing the surface with mannose to enable dual targeting of tumor cells and macrophages, which significantly enhanced the transfection and expression of mRNA encoding P53 (Figure 13B).^[^ ^155^ ^]^

## 5 Conclusion

mRNA therapy holds substantial promise in oncology, with its programmable design and rapid manufacturing capabilities offering innovative applications in tumor vaccines, protein therapies, cellular therapies, and gene editing. Despite its potential, several challenges remain, including mRNA stability, delivery efficiency, and immunogenicity, which must be effectively addressed to realize the full therapeutic potential. Advances in both viral and nonviral vector technologies are crucial for achieving precise and controlled mRNA delivery, thereby enhancing the safety and efficacy of cancer treatment. In developing delivery systems, achieving accurate cellular targeting, devising intelligent controlled‐release mechanisms, and optimizing administration routes are imperative. Moreover, stringent quality control measures, meticulous manufacturing management practices, robust funding mechanisms, and rigorous industry regulations must be implemented to facilitate clinical translation. The integration of cutting‐edge technologies, such as artificial intelligence and high‐throughput screening holds significant promise for accelerating the development of mRNA drugs. Consequently, mRNA therapy has the potential to significantly advance cancer therapy, fulfilling its clinical promise and providing new avenues for therapeutic intervention. The following section elaborates on the challenges and trends in mRNA‐based cancer therapy, exploring potential strategies.

### 5.1 Minimizing Immunogenicity and Cytotoxicity of Delivery Systems

The transition of mRNA therapeutics from the laboratory to clinical settings demands overcoming regulatory and translational challenges to ensure their safety and efficacy in a clinical context. Despite extensive testing, ongoing preclinical and clinical studies will evaluate potential safety concerns, including the induction of local and systemic inflammation, autoimmune diseases, and the potential toxicity of exogenous nucleic acids or delivery vehicle components.^[^ ^156^ ^]^ Therefore, toxicological assessments ensure that mRNA delivery vehicles comply with regulatory standards.^[^ ^157^ ^]^ For instance, the toxicity of lipids and PEG has raised concerns,^[^ ^158^ ^]^ and repeated dosing leading to lipid accumulation in tissues may pose safety risks, such as cellular and/or genotoxicity. Consequently, developing more biocompatible and stable particles, such as exosomes, polymer/lipid nanoparticles, safer biodegradable lipids, and hydrophilic materials as alternatives to PEG, represents an emerging trend.

### 5.2 Enhancing the Targeting Precision and Controllability of Delivery Systems

The specificity of mRNA‐based drugs has been attributed primarily to advances in targeted delivery systems. Strategies incorporating active targeting, passive targeting, and controlled release mechanisms have been developed. However, it is crucial to acknowledge the limitations imposed by tumor heterogeneity on receptor‐mediated active targeting, the ambiguity of structure–activity relationships in passive targeting, and the challenges of signal‐responsive controlled release systems in addressing early‐stage tumors and metastatic niches. Looking ahead, the evolution of cutting‐edge technologies, including machine learning, extensive data analytics, barcode methodologies, combinatorial chemistry, and high‐throughput screening, is poised to provide innovative approaches and novel perspectives for the development of novel formulations, materials, and a deeper understanding of the structure–activity relationship within nanoparticles. These advancements are expected to have a significant impact on the field by facilitating the design of more effective and safe mRNA therapeutics.

### 5.3 Developing the Direct Cytoplasmic mRNA Delivery Strategies

Direct cytoplasmic delivery is crucial for optimizing the efficacy of mRNA therapeutics, particularly in scenarios where the efficient in vivo production of engineered cells is essential. Innovations such as cell membrane fusion strategies have been devised to enhance the transfection efficiency of CAR mRNA in vivo. For example, Zhuo et al. discovered that cholesterol‐rich exosomes facilitate membrane fusion for the cytoplasmic delivery of nucleic acids.^[^ ^159^ ^]^ In another instance, the fusion‐associated small transmembrane (FAST) family proteins are favored for their low molecular weight, single transmembrane span, and lack of complex structures. Brown et al. incorporated the FAST protein into proteolipid vehicles to facilitate vector‐cell membrane fusion and significantly enhanced cytoplasmic nucleic acid delivery efficiency.^[^ ^160^ ^]^ Additional strategies are anticipated to be developed in the future.

### 5.4 Combinatorial Therapeutic Strategies for Enhanced mRNA Delivery and Cancer Management

Recognizing the inherent limitations of single‐modality treatments or homogeneous delivery systems, recent studies have explored the synergistic potential of integrating traditional therapies, immunotherapies, cell therapies, and gene therapies. These approaches have been complemented by developing hybrid delivery vehicles composed of diverse materials, aiming to optimize mRNA delivery for more effective tumor treatment and prevention. Moreover, the combination of medical devices with pharmaceutical excipients has been empirically validated, as demonstrated by oral formulations incorporating microcapsules and inhaled formulations incorporating excipients. From a macroscopic perspective, the convergence of different therapeutic approaches and interdisciplinary collaboration is an evident and significant trend. As we look to the future, the myriad combinations of these strategies promise to generate intriguing research avenues. However, it is imperative to consider the complexity of these systems, as it may introduce increased uncertainty and novel challenges in the clinical translation of advanced therapeutic modalities. This necessitates a robust framework to navigate the intricacies of successful clinical implementation.

### 5.5 Enhancing mRNA Delivery Systems for Diverse Administration Routes

Currently, mRNA vaccines can be administered via multiple routes. The administration route markedly influences the absorption rate, efficacy, metabolism, and potential adverse reactions of mRNA therapeutics. In comparison to mRNA vaccines, mouse models in other applications (protein therapy) often necessitate 50–1000 times the mRNA dosage. However, prolonged administration can potentially activate the innate immune system, which may attenuate therapeutic protein expression. This demand for elevated protein expression has spurred the development of optimized mRNA sequences and advanced delivery vectors. Consequently, selecting the appropriate administration route is an additional strategy to augment protein yield with less mRNA dosage. This approach, however, introduces the reoptimization and redesign of delivery vectors to address those specific challenges effectively.

## Conflict of Interest

The authors declare no competing interests.

## Author Contributions

Z.Z. and Y.F. contributed equally to this work. Z.Z. was responsible for conceptualization, methodology, and visualization; wrote the original draft; and reviewed and edited the final manuscript. Y.F. authored the “cancer mRNA vaccines” section of the original draft and also reviewed and edited the final manuscript. S.J. contributed the sections on “inorganic delivery systems” and “biomimetic delivery systems” to the original draft. Y.M. wrote the “mRNA protein replacement therapy” section of the original draft. Y.Y. compiled the “chronology of pivotal milestones in the evolution of mRNA therapy” featured in the original draft. Q.L. authored the “cell therapy” section of the original draft. Y.L. penned the “gene therapy” section of the original draft. S.S. conceptualized the project, developed the methodology, supervised the work, secured funding, acquired resources, and reviewed and edited the final manuscript. J.W. led the conceptualization, methodology, and supervision, oversaw funding and resource acquisition, and reviewed and edited the final manuscript.
